# Excimer and Exciplex Formation in Gold(I) Complexes Preconditioned by Aurophilic Interactions

**DOI:** 10.1002/anie.201916255

**Published:** 2020-06-08

**Authors:** Hubert Schmidbaur, Helgard G. Raubenheimer

**Affiliations:** ^1^ Department Chemie Technische Universität München Lichtenbergstr. 4 85747 Garching Germany; ^2^ Department of Chemistry and Polymer Science University of Stellenbosch Private Bag X1 Matieland 7602 South Africa

**Keywords:** aurophilicity, excimers, exciplexes, gold, relativistic effects

## Abstract

Excimers and exciplexes are defined as assemblies of atoms or molecules **A**/**A**′ where interatomic/intermolecular bonding appears only in excited states such as [**A**_2_]* (for excimers) and [**AA**′]* (for exciplexes). Their formation has become widely known because of their role in gas‐phase laser technologies, but their significance in general chemistry terms has been given little attention. Recent investigations in gold chemistry have opened up a new field of excimer and exciplex chemistry that relies largely on the preorganization of gold(I) compounds (electronic configuration Au^I^(5d^10^)) through aurophilic contacts. In the corresponding excimers, a new type of Au⋅⋅⋅Au bonding arises, with bond energies and lengths approaching those of ground‐state Au−Au bonds between metal atoms in the Au^0^(5d^10^6s^1^) and Au^II^(5d^9^) configurations. Excimer formation gives rise to a broad range of photophysical effects, for which some of the relaxation dynamics have recently been clarified. Excimers have also been shown to play an important role in photoredox binuclear gold catalysis.

## Graphical abstract

**Bond formation through excitation**: Linear, two‐coordinate gold(I) complexes are known to aggregate side‐on through aurophilic interactions. The resulting weak Au–Au contacts are strengthened upon UV irradiation as evidenced by a shortening of the intermetallic distance and the vibrational and electronic absorption and emission characteristics of the resulting excimers. The changes in the redox potentials make [Au^I^−Au^I^]* excimers useful intermediates in photoredox catalysis.

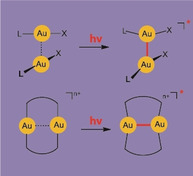

## 1 Introduction

The structural chemistry of gold(I) is largely characterized by its low coordination number (CN=2) with two tightly bound electroneutral (L) or anionic ligands (X) in the three combinations [L−Au^I^−L]^+^, [L−Au^I^−X], and [X−Au^I^−X]^−^ with linear geometry (**A**–**C** 
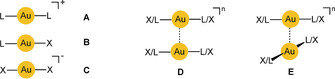
). Although this arrangement still gives free access to the central gold atoms, these metal centers are poor electrophiles, and only the strongest “soft” donors are accommodated to form complexes with CN>2. Bending of the L/X−Au^I^−L/X axes to accommodate an additional ligand is associated with a significant energetic cost, difficult to compensate by the energy of formation of a third or fourth Au^I^−L/X bond.^1^, ^2^, ^3^, ^4^, ^5^ The preference for CN=2 of the gold(I) centers is due to strong relativistic effects, which lead not only to a contraction of the atomic/cationic radii, but also modify the orbital characteristics of the cation and favor hybrids with high s‐character for bonding.^6^, ^7^, ^8^, ^9^

Conversely, the linear two‐coordination of gold(I) allows for a close mutual approach of its complexes to establish short Au^I^⋅⋅⋅Au^I^ contacts (shown for L−Au−X in **D** and **E**, with *n*=2− to 2+ 
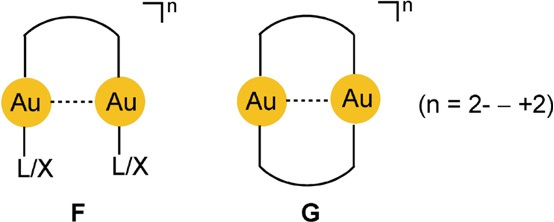
). Even with bulky ligands L/X, close Au⋅⋅⋅Au contacts are possible owing to the freedom of mutual rotation of the two molecular axes to give any conformation between eclipsed and staggered. Notably, through these Au⋅⋅⋅Au contacts the linearity of the L/X−Au−L/X axes is not strongly affected. In di‐ or multinuclear gold(I) complexes, the Au⋅⋅⋅Au contacts can be supported by bridging difunctional donor ligands, which leads to open‐chain or cyclic arrangements with geometrical details largely determined by the rigidity of the ligand connectors. Conformational changes are induced that allow for ring closure via the Au⋅⋅⋅Au contact (**F**) or enhance transannular Au⋅⋅⋅Au interactions (**G**).

The assumption of any significant Au−Au covalent bonding arising from the contacts between two neutral molecules [L−Au−X] is counterintuitive considering that the Au^I^ cations are in an electronic closed‐shell configuration (5d^10^) and have the same electrical charge (<+1), which arises from the polarization of the L−Au and Au−X bonds and should rather lead to Coulomb repulsion, leaving only dispersion forces as possible sources of very minor binding energies. However, a plethora of experimental evidence and the results of advanced quantum‐chemical calculations have shown that these Au^I^⋅⋅⋅Au^I^ interactions are indeed associated with energy contributions that exceed those of standard van der Waals interactions and may be comparable to those of other “weak interactions” including hydrogen bonding.^10^, ^11^, ^12^, ^13^ For gold, the phenomenon has been named “aurophilicity”, implying a direct attractive interaction between the Au atoms,^14^ but it has meanwhile been recognized that the term should be widened to “metallophilicity” to include most neighboring elements in the Periodic Table, preferably the heavy elements where relativistic effects also strongly influence the electronic configuration.^15^

The aggregation of [X−Au−X]^−^ anions or [L−Au−L]^+^ cations to give oligomeric anions {[X−Au−X]^−^}_*n*_ or cations {[L−Au−L]^+^}_*n*_ is even more intriguing as it is a unique case of “anti‐electrostatic association”, that is, it occurs against the Coulomb repulsion of ions. In this sense it resembles the “anti‐electrostatic hydrogen bonding” recently summarized for the anions HCO_3_^−^, HSO_4_^−^, and H_2_PO_4_^−^.^16^ This parallel also shows the comparable effects of the two weak forces hydrogen bonding and aurophilicity. Three specific examples of anti‐electrostatic aurophilicity are reviewed below (dicyano‐ and dithiocyanatoaurate(I) anions; di(ammine)gold(I) cations).

The fact that intramolecular aurophilic interactions may contribute significantly to the structural organization of di‐ or polynuclear gold(I) compounds, as well as to the assembly and packing of mono‐ and polynuclear gold(I) compounds in crystals and in solution, has many consequences for the properties of the various inorganic and organometallic systems. In recent years, mainly the solid state has been an area of widespread research activity regarding in particular the photophysical performance of gold(I)‐based materials.^17^ This work has provided early evidence that the emission properties show a dependence on the Au⋅⋅⋅Au distance in the crystals.^18^ The phenomena presently under investigation include many more diverse fields, such as thermo‐, photo‐, mechano‐, solvato‐, and vapochromism under standard or extreme pressures, where gold(I) compounds with intra‐ or intermolecular Au⋅⋅⋅Au contacts are found to show all kinds of multi‐stimuli‐induced light‐emitting properties.^19^, ^20^, ^21^, ^22^

**Scheme 1:**
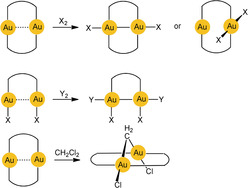
Oxidative addition reactions of cyclic and open‐chain dinuclear gold(I) complexes.

The chemical reactivity of gold(I) compounds is also strongly influenced by aurophilic interactions because they lead to arrangements that allow for direct di‐ or multicenter reaction pathways. Scheme 1 shows examples of transannular oxidative addition to dinuclear gold(I) complexes (d^10^–d^10^) affording long‐sought gold(II) complexes (d^9^–d^9^) or products with mixed oxidation states (X, Y=halogen; X_2_=disulfide RSSR; CH_2_Cl_2_).^23^, ^24^, ^25^

For mononuclear gold(I) complexes, related phenomena in solution are rare because the weak aurophilic interactions can easily be overruled by efficient solvation of the mononuclear units. However, at high concentrations of the complexes, and at low temperatures, the number of aurophilicity‐based dimers or oligomers is increased, and the association can be detected by various analytical techniques and in particular by following the UV/Vis absorption and emission properties.^26^ It is important to remember in all discussions that Au⋅⋅⋅Au interactions—as weak forces—quite generally make only small contributions to the overall energetics of a given system and that only a careful evaluation of all parameters can provide a consistent picture of the true origin of related chemical and physical phenomena. Recent publications refer to these inherent problems^27^, ^28^ that had been pointed out also in most previous Reviews of the subject.^11^, ^12^, ^13^, ^14^

As expected, intermolecular aurophilic contacts are even more pronounced for aggregates of di‐ or polynuclear gold(I) complexes where more than one Au⋅⋅⋅Au contact can be established. Early and more recent examples reflect the growing evidence for these interactions (**H** 
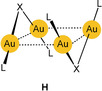
: X=Cl, Br; L=PR_3_; overall charge 2+).^29^, ^30^

**Scheme 2:**
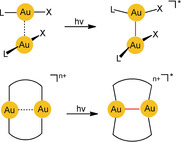
Photochemical generation of excimers of mono‐ and dinuclear gold(I) complexes.

In the present Review, we direct the reader to the specific phenomenon of [Au⋅⋅⋅Au] excimer and exciplex formation, which is strongly supported by an aurophilicity‐based preorganization of the components. In these [Au−Au]* excimers, which represent excited states of the aurophilic assembly, the two gold atoms are more tightly bound to each other than in the ground‐state structures of the precursor assemblies (Scheme 2; *n*=0 or 2). Red lines were introduced in the formulae here and below to highlight excimer bonding—the focus of this Review.

Surprisingly, this phenomenon has been followed up only sporadically in experimental and theoretical studies, and the results have not received much attention even though early examples were first published already some 25 years ago. [Au−Au]* bonding in the excimers is an interesting intermediate between strong ground‐state Au^0^−Au^0^ and Au^II^−Au^II^ bonding on the one hand and weak aurophilic interactions Au^I^⋅⋅⋅Au^I^ on the other. The incentive for the work carried out in recent years arose from the early observations and studies by the groups of Fackler, Che, and others, but most of the long‐term advances were achieved by the groups of Patterson and Omary, complemented more recently by theoretical and experimental studies by the groups of Yam, Iwamura, Kim, Thiel, Yersin, and several others as presented below.

To set the stage for this small treatise, in a first introductory section the established cases of “true” Au−Au single bonding with high covalent character (5d^10^6s^1^–5d^10^6s^1^ for Au^0^, and 5d^9^–5d^9^ for Au^II^) are presented, which provide the necessary reference data for bond lengths and energies and their variation with external parameters. This is followed by a brief summary of the corresponding data accumulated for inter‐ and intramolecular aurophilic interactions, which depend not only on the ligands X and L and their substituent patterns, but again also on external influences. In the final and most topical chapter, presently available data on gold(I) excimers and exciplexes are presented and discussed in this general context.

Owing to the absence of similar phenomena in compounds of Au^III^ (d^8^), this higher oxidation state was not considered. The enormous complexity of the photophysical properties of gold clusters and nanoparticles—with an average oxidation state of the cluster gold atoms between Au^0^ and Au^I^—was also beyond the scope of the present account, which tries to illustrate the excimer/exciplex effects in simple examples.

## 2 Benchmark Data for Gold–Gold Bonding in Dinuclear Au^0^ and Au^II^ Compounds

### 2.1 The Gold–Gold Bond in the Au_2_ Molecule and Its Complexes

Diatomic gold molecules Au_2_ are components of gold vapor generated from gold metal (b.p. 3080 K) upon heating in furnaces to above 2000 °C or by laser ablation and vaporization. Gas‐phase spectroscopic studies of the Au_2_ dumbbell molecule have a long history. Its molecular constants have been determined by various spectroscopic techniques, including resonant two‐photon ionization and supersonic molecular beam studies. From rotationally resolved spectra, the experiments finally arrived at an Au−Au distance for the ground state of 2.4715 Å and a dissociation energy of 53.0 kcal mol^−1^.^31^, ^32^, ^33^, ^34^, ^35^

**Scheme 3:**
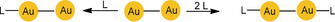
The gold molecule Au_2_ and its complexes.

This distance is surprisingly sensitive to the influence of ligands as demonstrated by far‐IR resonance‐enhanced multiple photon dissociation spectroscopy and by quantum‐chemical calculations. This is true even for the 1:1 and 1:2 adducts with noble gases, for example, for the recently studied adducts Au_2_⋅Kr and Au_2_⋅Kr_2_ (Scheme 3, L=Kr). In these linear molecules (C_*∞ν*_, D_*∞h*_ symmetry), the Au−Au distances have been calculated (MP2) to be slightly shortened from 2.429 (for Au_2_) to 2.421 and 2.40 Å (for Au_2_⋅Kr and Au_2_⋅Kr_2_, respectively).^36^, ^37^, ^38^

Attempts to obtain stable complexes of Au_2_ were unsuccessful for a long time,^39^, ^40^ until the group of Bertrand achieved the synthesis of a bis(carbene) complex in 2013.^41^ Using their powerful CAAC ligands, stable, crystalline complexes (CAAC)Au−Au(CAAC) were obtained, and the molecular structures could be determined (Scheme 3, right: L=CAAC). Surprisingly, the Au−Au distance was found to be 2.5520 Å long. Quantum‐chemical calculations performed in this work (EDA‐NOCV) resulted in Au−Au distances for the free Au_2_ molecule and its complex of 2.546 and 2.579 Å, respectively, indicating a slight weakening of the Au−Au bond upon complexation. For the as yet inaccessible phosphine complex (H_3_P)Au−Au(PH_3_), a distance of 2.550 Å has been predicted, and 2.562 Å for the Au_2_ molecule (MP2).^40^ For Au_2_ there is obviously a significant difference between these calculated values and the experimental value of 2.4715 Å (see above). Earlier^42^, ^43^ and more recent theoretical studies^37^, ^38^ of Au_2_ have arrived at data near 2.50 Å (MP2) and at the shorter end again (MP2: 2.429 Å).^37^, ^42^, ^43^, ^44^ This discrepancy is currently unexplained.

The Au_2_ molecule has recently also become a model system for “regium bonds” at gold clusters. In these studies, the surfaces of Au_*n*_ nanoparticles are analyzed to localize areas showing a preference for donor and acceptor interactions with substrate molecules.^45^ The diatomic molecule is the smallest “cluster”, and it has been found that it offers electrophilic “σ‐holes” at the extensions of the molecular axes for the docking of nucleophiles such as ammonia or water,^44^, ^46^ distantly resembling active sites on a clean gold surface.

### 2.2 The Gold–Gold Bond in Dinuclear Gold(II) Complexes

**Scheme 4:**
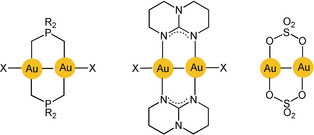
Transannular Au−Au bonding in cyclic dinuclear gold(II) complexes.

Au^II^−Au^II^ bonding was first established by transannular addition of oxidants to dimetallacycles with two gold(I) centers. The ligand frame was built using the triatomic bridges of phosphonium‐bis‐methylides^47^, ^48^ or diphosphinomethanes,^49^, ^50^ which provide C‐P‐C and P‐C‐P linkages, respectively (**G** and Scheme 1). Later work has also turned to ligands with P‐C‐S linkers^51^ or combinations with dithiocarbamates.^52^, ^53^ Further extensions used P‐C‐C bridging by aryl phosphines for a variety of mono‐ and polycyclic variants.^54^, ^55^ The most recent variations included metallacycles with N‐C‐N clamps based on amidinates and guanidinates (Scheme 4).^56^, ^57^, ^58^ Finally, difunctional carbene ligands were introduced as ligands of cyclic dinuclear complexes, albeit with larger ring sizes.^59^ A unique case was provided by the group of Wickleder, who prepared the gold(II) sulfate AuSO_4_. Crystals of this compound feature an eight‐membered ring with two sulfates bridging the two gold(II) centers.^60^ Its gold atoms are coordinatively saturated by sulfate O atoms of neighboring molecules.

As already shown in Scheme 1, the molecular halogens Cl_2_, Br_2_, I_2_, and some of their interhalogen compounds, alkyl halides, or disulfides (RSSR) were employed as oxidants. In the oxidative addition, the size and shape of the metallacycle remain largely unchanged, but the originally linear, two‐coordinate gold(I) centers are converted into gold(II) centers with a square‐planar coordination environment, and the transannular Au⋅⋅⋅Au distance is decreased significantly (see below). The newly formed Au−Au bond is thus doubly embraced by two ligand clamps. Recent studies have also provided access to cyclic Au^II^−Au^II^‐bonded compounds by electrochemical approaches.^61^

**Scheme 5:**
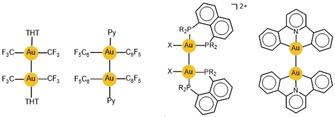
Au−Au bonding unsupported by ligands in dinuclear gold(II) complexes.

Compounds with Au^II^−Au^II^ bonds not embraced by ligands were found much later and often obtained unexpectedly, for example, in Au^I^/Au^III^ comproportionation reactions. Early examples were reported by the groups of Yam in 1996^62^, ^63^ and Kuz′mina in 2003,^64^ followed by those of Raubenheimer,^65^ Mathur,^66^ and Bochmann.^67^, ^68^ Theoretical work by Pyykkö has confirmed the thermodynamic stability of the unsupported Au^II^−Au^II^ bond as a sole linkage between two molecular units.^69^ Examples are shown in Scheme 5.

The Au−Au distances in the diauracyclic compounds (Scheme 4) span a narrow range, with lower limits of 2.47 and 2.49 Å for the guanidinates and the sulfate with small nitrogen or oxygen donor atoms, respectively,^57^, ^60^ and an upper threshold of 2.61 Å for the ligands with larger sulfur or selenium donor atoms (2.61 and 2.72 Å)^51^, ^52^ or for macrocyclic systems with unfavorable conformations.^59^

For the unsupported examples (Scheme 5), the range is similar and surprisingly narrow as it lies between 2.49 and 2.61 Å for complexes with a C^N^C pincer ligand^67^ and a 1,8‐diphosphinonaphthalene ligand,^62^ respectively. DFT calculations by Xiong and Pyykkö predicted Au^II^−Au^II^ distances of 2.50–2.55 Å for dinuclear model compounds related to the corresponding molecules used in the experimental work,^66^ with a bond energy of approximately 47.8 kcal mol^−1^.^69^

## 3 Relevant Aspects of Aurophilic Au^I^−Au^I^ Bonding

### 3.1 Background

The prototypes of aurophilic bonding were presented in Section 1 (**A**–**F**).^1^, ^70^ In all cases, gold atoms are drawn together to sub‐van der Waals distances whenever this approach is allowed by the steric properties of the system. This attraction also leads to the unforced, counterintuitive gathering of complex gold(I) units [(L)Au]^+^ at a given main‐group‐element or transition‐metal coordination center E in cations {E[Au(L)]_*n*_}^*m*+^.^71^, ^72^, ^73^, ^74^, ^75^

As a first criterion for further characterization, molecular and crystal structures were analyzed in order to determine the relevant range of Au–Au distances. Surprisingly, for gold(I) complexes the lower end of about 2.75 Å is already shorter than the sum of two metallic radii (2.88 Å in fcc gold metal) and not much larger than the distances found in diamagnetic dinuclear complexes of gold(0) (5d^10^6s^1^) and gold(II) (5d^9^), which both feature a covalent Au−Au bond (2.47–2.61 Å; see above). At the upper end, significant attraction is still observed (and calculated) near 3.50 Å, which is close to the sum of two van der Waals radii of gold(I) (ca. 3.60 Å).

As another structural criterion, the directionality of the bonding was found to be rather unspecific as suggested by the flexibility of the relevant bond angles and torsional angles involving Au–Au contacts. Thirdly, a given gold(I) center can entertain more than one Au⋅⋅⋅Au contact and may become part of oligomeric and polymeric aggregates where the monomers are tied together by aurophilic interactions with and without ligand support.

All of these structural observations have been confirmed by quantum‐chemical calculations on various levels of sophistication, which were aimed foremost at an estimation of the interaction energy as the most relevant criterion. Calculated energy profiles of the approach of two mononuclear model molecules L−Au−X exhibit shallow, but distinct minima for Au–Au distances in the range mentioned above and are largely insensitive to torsional constraints as exemplified by parallel (eclipsed) or perpendicular (staggered, “crossed swords”) conformations, the latter being only slightly preferred over the former (**D, E**). The interaction energies depend strongly on the nature of the ligands, but may reach up to 15 kcal mol^−1^.^76^, ^77^, ^78^, ^79^, ^80^, ^81^, ^82^, ^83^, ^84^

These theoretical results are in agreement with the few experimental data obtained in a series of variable‐temperature NMR studies, which cluster between 5 and 15 kcal mol^−1^.^85^, ^86^, ^87^ In theoretical calculations, the model {HAu(PH_3_)}_2_ dimer was shown to have a Au–Au distance of 3.0 Å and a bond energy of 13.0 kcal mol^−1^.^88^

This energy criterion indicates that aurophilic bonding ranks among the weak forces in chemistry, and the closest parallels are found for hydrogen bonding, where interaction energies are in a comparable range. As already mentioned, both hydrogen bonding and aurophilic interactions show a similar structural flexibility regarding contact distances and angles. There is also a degree of freedom in the number of contact partners, meaning in the aurophilicity case that several Au⋅⋅⋅Au contacts can be formed by the reference gold atom in larger aggregates (see above).

### 3.2 Intermolecular Aurophilic Contacts between Mononuclear Gold(I) Complexes

While for the solid state there are a plethora of examples for the aggregation of mononuclear gold(I) complexes through aurophilic interactions (see above), the situation is different for the solution state, where molecular or ionic units **A**–**C** are enclosed by solvent molecules and the energy of solvation exceeds the energy gained from aurophilic contacts. For that reason, in dilute solutions of electroneutral molecules **B** in nonpolar or weakly polar solvents, the molecules are not found aggregated as **B**_2_ or **B**_*n*_ oligomers, as shown for example, by standard molecular mass determination (cryoscopy, ebullioscopy etc.) or mass spectrometry studies.

However, with the removal of the solvent, the organization of the electroneutral components **B** in the crystals is co‐determined by aurophilic interactions, which are stronger than common van der Waals forces, as a directional element for the assembly. The aggregation of ionic counterparts **A** and **C** is largely governed by Coulomb forces favoring sequences of alternating charges +−+−+− (**A**⋅⋅⋅**C**⋅⋅⋅**A**⋅⋅⋅**C**⋅⋅⋅**A**⋅⋅⋅**C**). Other interactions such as π–π stacking or strong hydrogen bonding may also influence the mode of aggregation ruling out any Au⋅⋅⋅Au contacts. On the other hand, there are many cases where aurophilic and hydrogen bonding lend mutual support to a structure of di‐ or multinuclear aggregates.^89^, ^90^ In the following, the aurophilicity performance of mononuclear gold(I) complexes is illustrated for three examples most relevant in the present context, that is, for the formation of excimers.

#### 3.2.1 The Case of the Dicyanoaurate(I) Anion

**Figure 1:**
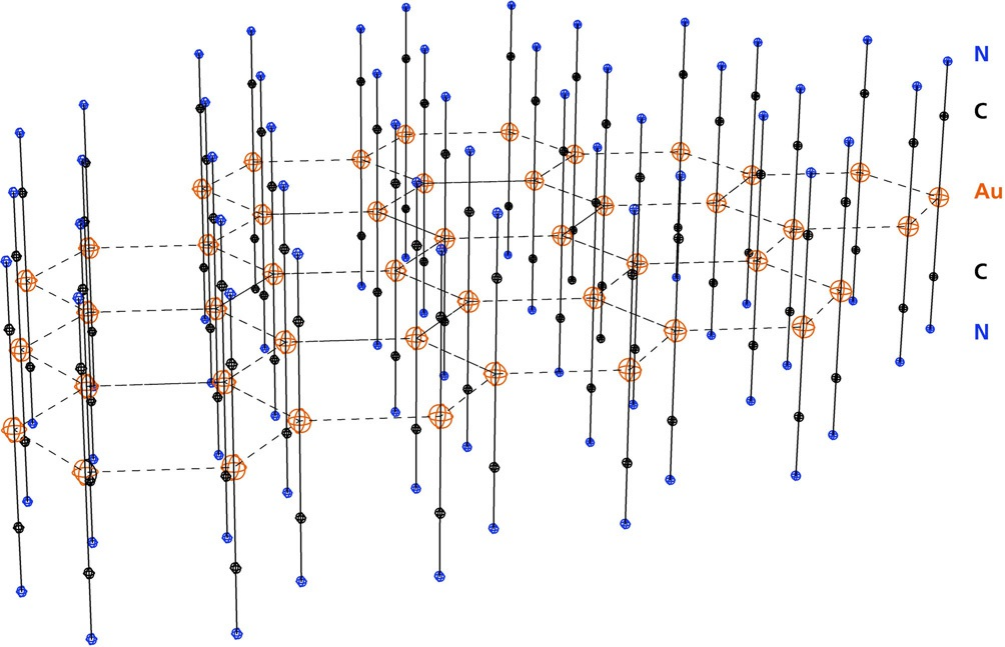
Idealized structure of a {[Au(CN)_2_]_*n*_}^n−^ polyanionic sheet present in the crystals of most alkali and alkaline earth metal dicyanoaurates(I).

An important and most widely studied example is the (anti‐electrostatic^16^) aggregation of the dicyanoaurate(I) anion [NC−Au−CN]^−^ in crystals and solutions of the salts M^+^[Au(CN)_2_]^−^, M^2+^{[Au(CN)_2_]_2_}^2−^, and M^3+^{[Au(CN)_2_]_3_}^3−^. In crystals of the alkali salts (M=Na, K, Rb, Cs), the anions are packed in sheets with all gold atoms in a common plane and with the cyanide groups extending roughly perpendicular from this plane above and below. The assembly pattern depends on the size of the counterion, and the Au⋅⋅⋅Au contacts vary across a small range around an average of 3.33 Å.^91^, ^92^, ^93^, ^94^, ^95^, ^96^ Figure 1 shows an idealized structure of a {[Au(CN)_2_]_*n*_}^*n*−^ sheet with the most common hexagonal array of gold atoms. As an example of a distorted variant, in crystals of Rb[Au(CN)_2_], the gold atoms are arranged in a plane composed of four‐ and eight‐membered rings.^96^

Already in many publications of early structural work it has repeatedly been assumed that “*there may be significant bonding interactions among the gold atoms in these solids*”.^97^ Such “*low‐dimensional Au⋅⋅⋅Au interactions*” have also been held responsible for the unusual absorption and emission characteristics of alkali dicyanoaurates(I) under high pressures (20 kbar). Extreme red‐shifts of the emission were observed, which are among the largest known for solid‐state compounds.^98^ Anomalies were also observed for crystalline powders of K[Au(CN)_2_] in ^197^Au Mössbauer spectroscopic investigations at low temperature (4 K) and high pressure (80 kbar), which suggested a preference for Au⋅⋅⋅Au contacts in the packing.^99^

Pioneering work has also included salts with bulky cations, as in NBu_4_[Au(CN)_2_], which separate the anions in the crystals^100^ such that the photophysical characteristics of isolated [Au(CN)_2_]^−^ anions could be studied in the same way as in crystal matrices doped with traces of M[Au(CN)_2_].^101^, ^102^, ^103^ In crystals of salts with very bulky cations such as bis(triphenylphosphoranylidene)ammonium [(Ph_3_P)_2_N]^+^ also no aggregation of the anions occurs.^104^

Early studies of the vibrational spectra of dicyanoaurates(I) provided no evidence for a specific influence of the Au⋅⋅⋅Au contacts on the fundamental characteristics of the anion in solids, and solution IR and ^13^C NMR spectra (with ^13^C‐labeled cyanide) at various concentrations (in water and dichloromethane at room temperature) did not indicate any major effects of anion aggregation.^105^

As already mentioned, the luminescence properties of alkali metal dicyanoaurate(I) solids have been in the focus of photophysical studies for several decades.^106^, ^107^ Most work also included the silver analogues for comparison.^107^ These studies have led to the consideration of [Au−Au]* excimers and exciplexes and a detailed study of their characteristics. Several recent quantum‐chemical calculations have followed up the early experimental work and detailed the picture of the electronic ground‐ and excited‐states, in particular of the dimers and trimers (**I**, **J** 
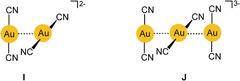
), as presented below.^108^ The structural dynamics of the latter were finally followed by picosecond/femtosecond time‐resolved emission and absorption spectroscopy^109^, ^110^ and femtosecond X‐ray solution scattering.^111^, ^112^, ^113^

Investigations of aqueous solutions or in polar non‐aqueous solvents have shown that therein the degree of oligomerization increases stepwise with the [Au(CN)_2_]^−^ concentration, which allows for dimers, trimers, and tetramers to be studied in separate concentration ranges (e.g., 30 mm for the dimer, 300 mm for the trimer in water) and their formation and transformation to be followed. This association explains the observation of significant deviations from Beer's law in the spectra of these solutions. The absorption and luminescence excitation spectra of water or methanol solutions also show a dependence on temperature from ambient temperature to 77 K (in frozen glasses). As expected, an increase in the aggregation of the anions to oligomers was found at low temperature. From the spectral data, both the formation constant and the free energy of formation have been calculated for the dimer: *K*=17.9 m ^−1^; Δ*G*=−1.86 kcal mol^−1^ (295 K). Extended Hückel calculations with relativistic parameters for the dimer (eclipsed/staggered) arrived at Au⋅⋅⋅Au binding energies of 3.04/6.87 kcal mol^−1^ and Au–Au distances of 3.48/2.88 Å.^101^, ^107^ Further luminescence studies, which are much more sensitive than absorption studies, have shown that oligomerization occurs even at concentration as low as 10^−5^ m (observed for methanol glasses at 77 K). For the dimer in a staggered conformation, MP2 calculations gave an Au–Au distance of 2.960 Å and a ν(Au⋅⋅⋅Au) stretching frequency of 89.8 cm^−1^.^107^ Later, MP2 calculations of models of the dimer yielded Au–Au distances of 3.014 and 2.998 Å for the staggered dimer and trimer, respectively.^108^

The aggregation of dicyanoaurate(I) anions was recently also observed in ionic liquids. Time‐resolved luminescence spectra revealed the presence of different oligomers both in imidazolium and pyrrolidinium salts.^114^

If the counterion of the dicyanoaurate(I) anion contains a two‐coordinate gold(I) atom, for example, with the bulky PTA ligand (1,3,5‐triaza‐7‐phosphaadamantane) in [(PTA)Au(PTA)]^+^, then the aggregation of the ions follows the sequence +−+−+− with alternating aurophilic contacts between cations and anions, and no [Au(CN)_2_^−^]_*n*_ oligomers are formed. The slim, rod‐like structure of the anions fits even into the reduced space left between the bulky PTA ligands to establish short Au⋅⋅⋅Au contacts (3.271 Å).^115^

In this context, the design of systems where certain oligomeric units [Au(CN)_2_^−^]_2_ are trapped in supramolecular matrices offered additional means to distinguish the specific characteristics of the dicyanoaurate(I) oligomers.^116^ With 24‐pyrimidinium crown‐6 and 16‐pyrimidinium crown‐4 polycations, the dimers, trimers, tetramers as well as chain‐like aggregates, in all‐staggered conformations, appear in the voids of the crystalline supramolecular assemblies.^117^ A recent example has been provided by the reaction of the viologen 1,1′‐bis(2,4‐dinitrophenyl)‐4,4′‐bipyridinium dichloride ((DNP)Cl_2_) with K[AuCN)_2_] in water, which gave the crystalline tetrahydrate (DNP)[Au(CN)_2_]_2_⋅4 H_2_O. The product contains dinuclear units {[Au(CN)_2_]_2_}^2−^ with an eclipsed (parallel) conformation with a Au⋅⋅⋅Au contact at the upper limit for aurophilic interactions (**D**, *d*(Au⋅⋅⋅Au)=3.5108 Å), in agreement with a calculated value for this conformation, which is not the ground state in the absence of a matrix.^101^, ^107^ While (DNP)Cl_2_ and [Au(CN)_2_]^−^ are non‐emissive in the visible region, (DNP)[Au(CN)_2_]_2_ shows two independent emission bands in the regions assigned to the DPN and the [Au(CN)_2_^−^]_2_ units, the former being a phosphorescence due to the interionic interaction of DPN with the heavy metal atoms.^118^

Polymeric strands [Au(CN)_2_^−^]_*n*_ can be supported by polyammonium scaffolds such as those provided by polyallylamine hydrochloride polymers, which undergo an ion exchange of [Au(CN)_2_]^−^ for Cl^−^ in water. The aggregates show tunable luminescence depending on the relative concentrations of the components. With a low supply of anions, their chains may be broken down into smaller oligomers. This process can be followed by the changes in the absorption and emission spectra with concentration.^119^ Using a polyrotaxane host for [Au(CN)_2_]^−^ anions has afforded crystalline products in which the anion is associated in symmetrical trimers (**J**) with Au–Au distances of 3.103 Å and a Au‐Au‐Au angle of 180°.^120^ This result is also in good agreement with calculated data.^101^, ^107^ In contrast, with an aza[5]helicene viologen acceptor, dimers [Au(CN)_2_^−^]_2_ with an eclipsed conformation (*d*(Au⋅⋅⋅Au)=3.310 Å) were again found in the crystalline matrix.^121^ All of these examples demonstrate the important influence of independent cations on the one hand and of structured cationic matrices on the other hand on the resulting mode of anion aggregation.

The structural and electronic characteristics of crystalline dicyanoaurates(I) are strongly influenced by cations such as thallium or platinum, which appear to form heterometallophilic contacts, as in Tl[Au(CN)_2_]^122^, ^123^ and [Pt^II^(NH_3_)_4_][Au(CN)_2_]_2_.^124^ By contrast, in the Ni^II^ complex [Ni(NH_3_)_2_][Au(CN)_2_]_2_, the chains of [Au(CN)_2_]^−^ anions do not include the nickel atoms. These differences can again be ascribed to relativistic effects, setting, for example, Pt apart from its lighter congener Ni.^125^

Extended aggregates {[Au(CN)_2_]^−^}_*n*_ appear in combination with first‐ and second‐row transition metals where the terminal N atoms of the cyanide units act as donor centers for a large variety of elements, including Co, Rh, Cu, Cd, In, and others. Chains of oligomers and polymers [Au(CN)_2_]^−^}_*n*_ with the gold atoms forming angular or zigzag patterns are common in this family of structures.^126^, ^127^, ^128^, ^129^, ^130^, ^131^ The flexibility of the Au⋅⋅⋅Au contacts in crystals of these compounds allows for a rich dimensional flexibility, which becomes particularly obvious in anomalous (“colossal” or “giant”) positive and negative thermal expansion.^132^, ^133^ Early structure determinations of dicyanoaurates(I) of the rare‐earth elements also gave layered structures with the gold atoms arranged in Kagomé nets (three‐ and six‐membered rings) with Au−Au distances of 3.316 Å. Surprisingly, the NC−Au−CN anions were found to be strongly bent, which allowed for both short Au⋅⋅⋅Au contacts and an optimized prismatic coordination of the cations.^134^ In another series of studies, the structures and luminescence properties of rare‐earth dicyanoaurates(I) were again investigated, revealing examples where aurophilic contacts are present or absent. In the crystals, the energy transfer in the former clearly occurs through the Au⋅⋅⋅Au contacts.^135^ This work has also been extended to include studies of aqueous solution systems.^136^

In a discussion of the oligomerization of dicyanoaurate(I) anions, its role in the technology of gold leaching and refining in the carbon‐in‐pulp process must be mentioned.^137^ For over a century, it has been known that dicyanoaurate(I) anions contained in aqueous solutions are strongly adsorbed on the surface of active carbon, from where it can be desorbed and recovered by caustic or caustic cyanide solutions at elevated temperatures. The loaded carbon, which may contain between 200 and 20 000 grams of gold per ton is indicative of a very close aggregation of the anions on the surfaces, probably as dimers.^138^, ^139^ Notably, other complex salts of gold(I) or gold(III) do not show a similar affinity to carbon surfaces. Obviously, the linear five‐atomic coordination geometry not only offers ready access for aurophilic contacts, but also optimal conditions for adsorption to suitable surfaces.

#### 3.2.2 The Case of the Di(thiocyanato)aurate(I) Anion

In this anion, the thiocyanato ligands are attached to the gold(I) center via their sulfur atom, and the complex [Au(SCN)_2_]^−^ has an angular structure bent at both sulfur atoms. In fundamental work in this area, the anions in crystals of bis(thiocyanato)aurates(I) with alkali or onium cations have been found by X‐ray diffraction studies to form dimers {[Au(SCN)_2_]^−^}_2_ almost exclusively through anti‐electrostatic aurophilic contacts. The two anions of *C*_2_ symmetry are associated in a staggered conformation, and the line connecting the two gold atoms coincides with the twofold axis (**K** 
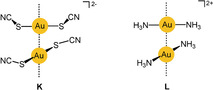
). The Au–Au distances vary with the nature of the counterion, but all fall in the narrow range of 3.00–3.24 Å. Interestingly, this also applies to the [N^*n*^Bu_4_]^+^ salt, where the Au–Au distance in the dimer is 3.07 Å. It is only in the [NMe_4_]^+^ salt that the anions are aggregated into trimers with a kinked structure (the Au‐Au‐Au axis being bent at the central gold atom). The maxima of the absorption and emission spectra of the compounds (solids at 77 K) vary with the nature of the cation, and the Au–Au distance correlates with the emission energy.^140^, ^141^

The mode of aggregation of the [Au(SCN)_2_]^−^ anions is an important example of the power of aurophilic interactions. The SCN^−^ anion has a hard (N) and a soft donor center (S) that could assist in the assembly of the anions via N→Au or S→Au donor bonds, but these alternatives are not observed. Instead, additional Au⋅⋅⋅Au contacts between the dimers can be found extending the arrangement to chains with a sequence of alternating Au⋅⋅⋅Au⋅⋅⋅⋅Au⋅⋅⋅Au⋅⋅⋅⋅Au distances.

In a subsequent study, the photophysical properties of the bis(thiocyanato)aurates(I) were investigated further to follow up on the intriguing structure–luminescence relationship.^142^ This work has led to the identification of the pertinent group of excimers **K** (see below).

#### 3.2.3 The Case of the Diamminegold(I) Cations [Au(NH_3_)_2_]^+^

To mention a cationic counterpart for the dicyanoaurate(I) anion, the mode of aggregation of the diamminegold(I) cation (**L**) in its salts is briefly introduced. The crystal structures of the chloride (as an ammonia solvate), bromide, nitrate, and perchlorate have been determined, and it has consistently been observed that the cations are associated into chains with Au⋅⋅⋅Au contacts of 3.176, 3.414, 3.091, and 2.990 Å, respectively. The change in distance with a switch in the anion is probably due to the support of the packing by hydrogen bonds both in the ammoniate of the chloride and in the nitrate and perchlorate. Crystals of the perchlorate show a strong and broad emission (450–650 nm) assigned to metal–metal charge transfer (MMCT) transitions.^143^, ^144^, ^145^

These three cases reflect both the long history and the current relevance of studies of intermolecular aurophilicity. Recent work has further increased the variability in the choice of ligands L and X for gold(I), to include also more and more exotic representatives, which determine their homo‐ and heteroleptic distribution and the length and strength of aurophilic contacts.^146^ In light of space limitations, these will not be discussed further.

### 3.3 Intramolecular Aurophilic Contacts in Di‐ and Polynuclear Gold(I) Complexes

The assignment of intramolecular aurophilic interactions between a pair or a larger number of gold(I) centers in a molecule (**F, G**) based only on Au^I^–Au^I^ distance criteria is often less straightforward than for the intermolecular cases as rigid ligand geometries may enforce certain configurations where the metal centers are held in a close proximity but which are not or not predominantly governed by aurophilic contacts.^147^, ^148^, ^149^ For ligands with a flexible geometry, the conformation **F** with an Au⋅⋅⋅Au contact found in a crystal may simply be determined by packing forces. For structures of type **G** a ring size of a minimum of seven atoms is required for strain‐free accommodation of two linearly coordinated gold atoms. For rings with seven to nine atoms, any transannular Au⋅⋅⋅Au contacts will be shorter than 3.5 Å. Therefore, in addition to single‐crystal X‐ray diffraction studies, the use of other analytical techniques is required for further characterization of Au–Au bonding, for example, in **F**‐ and **G**‐type molecules.

Early studies therefore concentrated on molecular dynamics. The vibrational frequency characteristics of intramolecular aurophilic contacts Au^I^⋅⋅⋅Au^I^ were first studied in a dinuclear gold(I) complex with two bridging deprotonated ylide ligands (**M** 
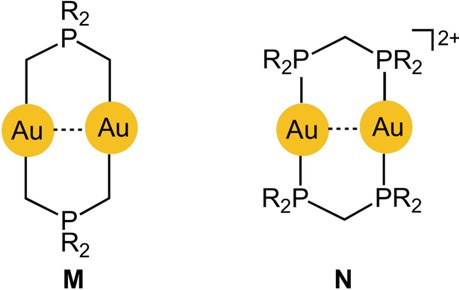
), and the result was compared with the data for the same dimetallacycle of gold(II) with a transannular Au^II^−Au^II^ bond generated upon oxidative addition of a halogen (X=Cl, Br, I; Scheme 1). Upon oxidation, the value of ν(Au⋅⋅⋅Au)=64 cm^−1^ was found to increase to 162, 132, and 103 cm^−1^, confirming the relative weakness of the aurophilic interaction.^150^ Similar values were later reported for dinuclear gold(I) dimetallacycles with diphosphinomethane ligands (**N**), and a correlation between the intramolecular Au−Au distances and the *F*(Au⋅⋅⋅Au) force constants was established. Structural parameters of the ground and excited states have been calculated (see also below).^151^, ^152^, ^153^ For the intermolecular aurophilic interaction between mononuclear gold(I) complexes, only data from quantum‐chemical calculations are available, which confirmed those of the dinuclear complexes.

A more recent report describes extended X‐ray absorption fine structure (EXAFS) investigations of a molecule with a flexible ligand carried out in solution in order to demonstrate that the conformation represented by **F** is retained in solution. For a selected group of compounds with bis(phosphine) ligands, the preference for the aurophilicity‐stabilized conformers was confirmed with the expected dependence on the nature of the solvent. For dinuclear, ligand‐bridged complexes, the Au–Au distances agree well with those found in the solid state, but the distances depend on both the counterion and the solvent. For [(dppm)_2_Au_2_]X_2_, the transannular Au–Au separations are 2.93 Å for X=Cl in the solid state and 3.08 Å for the solution in chloroform, while for X=BF_4_ the values are 2.90 Å in the solid and 2.97 Å in acetonitrile solution, that is, the solvent is lengthening and weakening the aurophilic interaction.^154^

Molecules with intramolecular aurophilic contacts (**F** 
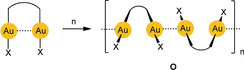
) may also be in equilibrium with oligomers tied up via intermolecular Au⋅⋅⋅Au contacts (**O**) depending on the solvent and pH value where the ligands may be deprotonated.^155^

**Scheme 6:**
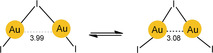
Bond bending isomerism in the Au_2_I_3_^−^ anion.

There are very few studies on intramolecular aurophilic bonding in the gas phase. A recently reported photoelectron spectroscopic study of the bonding in the Au_2_I_3_^−^ anion has presented a unique case of “bond‐bending isomerism”. For the V‐shaped pentaatomic anion of *C*_2*v*_ symmetry, an isomer with predominantly covalent bonding showing an obtuse Au‐I‐Au angle of 100.7° and a long Au⋅⋅⋅Au contact of 3.99 Å is in equilibrium with an isomer with an acute bond angle of only 72° and a short Au⋅⋅⋅Au aurophilic contact of 3.08 Å, which is only 0.425 kcal mol^−1^ higher in energy, that is, nearly degenerate (Scheme 6). At low temperature, only the obtuse isomer is observed. The anion is to be taken as an example of a molecule oscillating between different structures as a result of two competing chemical forces, as also supported by quantum‐chemical calculations.^156^ Larger [(AuCl)_*n*_Cl]^−^ anions have been assigned a zigzag structure with a central chain of gold atoms resembling that in crystals of AuCl.^157^

UV/Vis absorption and emission properties have been the main focus of studies of transannular Au⋅⋅⋅Au interactions.^17^, ^158^, ^159^, ^160^ Typical examples have been the eight‐membered‐ring complexes of 1,3‐difunctional phosphines where two roughly parallel P‐Au‐P axes are held in close proximity (**N**). A large number of experimental and theoretical studies were dedicated to this type of assembly.^161^, ^162^ In an early investigation, electronic configurations have been assigned to the ground and excited states, which allow an interpretation of the various transitions observed in solution. The excited state was found to be powerfully reducing with a redox potential of −1.6 V. Similar results were found for trinuclear analogues.^163^ Transannular aurophilic contacts have also been followed up in structural, spectroscopic, and theoretical studies of di‐ and trinuclear gold(I) complexes of a variety of diphosphine donors based on the xanthene skeleton, in recent cases also with dicyanoaurate(I) anions.^19^, ^20^, ^164^, ^165^

Work in this area was later complemented by experimental and quantum‐chemical studies of similar cyclic cationic complexes with P,S donor ligands as further detailed below.^166^ Even more intimate Au⋅⋅⋅Au contacts are established in N,N‐difunctional donor ligands, recently including di‐ and tetranuclear guanidinate complexes.^167^ In a similar way, phosphine donors have recently been combined with carbene donors (NHCs) to afford complexes with an eight‐membered ring or two fused eight‐membered rings with one or two transannular Au⋅⋅⋅Au contacts, respectively.^168^ All of these examples and a few more recent ones have served as ground states for the formation of the corresponding excimers as summarized in the following Sections.

## 4 Formation and Reactivity of and Bonding in [Au^I^ −Au^I^]* Excimers and Exciplexes

### 4.1 Background

About 100 years ago, the formation of spectroscopically observable diatomic molecules from electronically excited atoms was first achieved for gaseous mercury and the noble gases, which previously had been known as notoriously inert species.^169^, ^170^ Twenty years later, the formation of long‐lived excited dimers of pyrene and other aromatic organic compounds in solid and liquid phases elicited the interest of many scientists.^171^, ^172^ The term *excimer* was coined^173^ to distinguish such collisionally formed excited dimers from short‐lived, weakly associated dimers that exist in solution at relatively high solute concentrations or in the crystalline state. Excimer formation may be observed and studied experimentally by means of UV/Vis absorption, luminescence emission, time‐resolved absorption and emission, and resonance Raman spectroscopy. Time‐resolved solution X‐ray scattering has recently become another option, with exceptional advantages.

Finally, amongst compounds of the transition metals, and in particular those with Rh^I^, Pt^0^, Pt^II^, and Au^I^, structurally privileged complexes have also shown a tendency towards excimer formation.^174^ The monomers experience weak attraction in the ground state, which is now referred to as metallophilic contacts, mediating the formation of much stronger metal–metal covalent bonds in the excited state. In the presence of other suitably reactive ground‐state species, interactions with the excimers afford *exciplexes*. Oligomeric exciplexes, some originating from weakly associated ground‐state dimers or oligomers, are also known.^175^ Increasing numbers of complex excimers and exciplexes have been reported.^7^ Stimulus for the investigation into the excited state has been found in other photochemical and photophysical properties, such as thermochromism, optical memory, and tunability.

It therefore appears that covalent bonds formed by atomic orbital overlap between two gold atoms are not limited to Au^0^ and Au^II^ examples (see above): Two‐coordinate Au^I^ atoms can also become covalently bound exclusively in the excited state. Preorganization through aurophilic interactions [Au⋅⋅⋅Au] (the most prominent case of metallophilicity) in the ground state assists in the formation of Au^I^−Au^I^ chemical bonding in the corresponding excimers, [Au^I^ −Au^I^]*, upon excitation.

According to a metal centered (MC) LCAO approach, metal–metal closed‐shell d^10^–d^10^ interactions of transition‐metal systems are repulsive. Yet, as already mentioned, metallophilic interactions of a dispersive nature tend to hold two gold atoms with this configuration together. Such interactions can only be accounted for, theoretically, by invoking excited‐state orbitals, that is, by using wave function methods in computational chemistry.^7^ Theoretically, the possibility of strengthening d^10^–d^10^ interactions by photochemical activation seems a logical consequence.

The idea that covalent bond formation in closed‐shell d^8^–d^8^ and d^10^–d^10^ systems can be realized by electron excitation is not new. It was proposed and explored by Gray and co‐workers during the 1970s and early 1980s for Rh^I^ and Pt^II^ complexes. Pairs of metal atoms bearing several monodentate ligands closely approach each other by metal–metal attraction in concentrated solutions,^176^, ^177^ or they are held in close proximity by double‐bridging diphosphine ligands.^178^ Experimental evidence for substantial Rh^I^ −Rh^I^ interactions in the excited state was obtained by solution resonance Raman spectroscopy.^177^

### 4.2 Excimer Bonding in Binuclear Au^I^ Complexes with Bridging Phosphine Ligands

#### 4.2.1 Early Experimental Studies and Interpretations

Inspired by the pioneering work of the Gray group, investigations led by Ludwig,^179^ Caspar,^180^ and again Gray^181^ into the excited states of double‐bridged dppm complexes of palladium, platinum, and gold opened new fields of research. The results were reported in quick succession in 1989–1993 in a number of important publications on the spectroscopic properties of complexes of the type [Au_2_(P^P)_2_]^2+^ (P^P=bis(dimethylphosphino)methane, dmpm; bis(dimethylphosphino)ethane, dmpe; bis(diphenylphosphino)methane, dppm); [Au_2_(P^P)_3_]^2+^ (P^P=dmpm); and [Au_3_(P^P^P)_2_]^3+^ (P^P^P=bis(dimethylphosphinomethyl)methylphosphine, dmmp).^158^, ^161^, ^163^, ^182^, ^183^, ^184^ The surprising photophysical properties reported for these examples, in which the metal atoms are held closely together by the ligands (formula **N**), provided the impetus for related research into excimer formation to become an independent worthwhile intellectual pursuit.

Early absorption and magnetic circular dichroism studies of the dinuclear dmpm and dmpe Au^I^ systems in solution showed that excitations resulting in luminescence are metal‐centered (MC).^161^, ^184^ In order to assign the absorption and most prominent luminescence bands, most of the early scientists, following Gray and co‐workers, relied on symmetry principles and electrostatic ligand‐field‐like considerations to construct molecular orbital (MO) energy diagrams in which the HOMO and LUMO orbitals were MC. The HOMOs, formed by linear combination mainly of 5d${}_{z{}^{2}}$ atomic orbitals of the two gold atoms (*z*‐axis along the Au–Au connectivity line in the weakly associated complexes) were designated dσ*. The LUMO orbitals, described as pσ, were formed by the combination of almost pure 6p_*z*_ atomic orbitals.

**Scheme 7:**
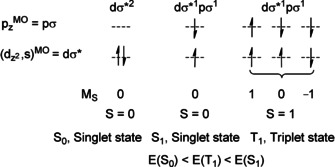
Microstates and term states for MO configurations dσ*^2^ and dσ*^1^pσ^1^ in Au_2_(P^P)_2_^2+^.

Fackler and co‐workers^158^ carried out SCF‐X_α_‐SW calculations for bis(diphosphinomethane) (dpm) model complexes, [Au_2_(H_2_PCH_2_PH_2_)_2_]^2+^ (**N**, R=H), with eight‐membered rings in the elongated chair conformation, with relativistic corrections applied. Unfortunately, this work failed to explain the Au^I^⋅⋅⋅Au^I^ association, and the relative energies of the unfilled orbitals were unreliable. Yet, the results agreed with more intuitive approaches. Despite the lack of solid theoretical guidance, the authors of these publications^158^, ^161^, ^163^, ^182^, ^183^, ^184^ reached consensus that the absorption of all of these complexes in the UV region can be assigned to MC, spin‐ and Laporte‐allowed dσ*→pσ transitions, with a one‐electron excitation involving two singlet states (Scheme 7). The emission (phosphorescence) in the visible region, exhibited by all of the complexes, was generally assumed to be mainly a pσ→d, a triplet–singlet transition. Che and Yam^163^, ^183^ briefly, on qualitative grounds, advocated the involvement of dγ*, i.e., d${}_{x{}^{2}-y{}^{2}}$ ‐based, MOs in the transitions, but later, with more experimental and theoretical evidence at their disposal, they accepted the general view of dσ*→pσ excitation and pσ→dσ* emission processes.

The one‐electron excitation from an antibonding to a bonding MO formally amounts to an increase in bond order of one, and the formation of a σ bond between the two gold atoms. The resulting molecular distortion (mainly the shortening of the Au–Au distance) is believed to be responsible for the large (ca. 17 000 cm^−1^) Stokes shift between the absorption and emission maxima.^158^

Though emission from the low‐lying ^3^[dγ*^1^pσ^1^] state is proposed again for the trinuclear complex [Au_3_(dmpm)_2_]^2+^ (in acetonitrile), this excited configuration still implies a Au^I^ −Au^I^ bonding situation with p_*z*_–p_*z*_ orbital overlap.^185^ An increase in the number of P_2_Au units results in a narrower dσ*–pσ energy gap while *E*(dσ*) increases; [Au_*n*+1_] units absorb at lower energy than [Au_*n*_] units.^163^

The relatively long lifetime of the triplet excited state was exploited by Che and co‐workers^161^, ^183^ in studying various chemical reactions during which the phosphorescence of the activated [Au_2_(dppm)_2_]^2+^ complex is quenched. In redox reactions (1), (2), *N*,*N*,*N′*,*N′*‐tetramethyl‐*p*‐phenylenediamine (TMPD) served as a reductant and *N*,*N′*‐dimethyl‐4,4′‐bipyridinium (methylviologen, MV^2+^) as an active oxidant:

(1) $$\left[\mathrm{Au}{}_{2}\left(\mathrm{dppm}\right){}_{2}{}^{2+}\right]{}^{*}+\mathrm{TMPD}\to \left[\mathrm{Au}{}_{2}\left(\mathrm{dppm}\right){}_{2}\right]{}^{+}+\mathrm{TMPD}{}^{+}$$

(2) $$\left[\mathrm{Au}{}_{2}\left(\mathrm{dppm}\right){}_{2}{}^{2+}\right]{}^{*}+\mathrm{MV}{}^{2+}\to \left[\mathrm{Au}{}_{2}\left(\mathrm{dppm}\right){}_{2}\right]{}^{3+}+\mathrm{MV}{}^{+}$$

The standard electrode potential of the activated couple [Au_2_(dppm)_2_^3+^]*/[Au_2_(dppm)_2_^2+^]* was estimated at −1.1 V against a saturated sodium chloride calomel electrode. Yam and co‐workers^163^ determined a value of −1.7 V for the redox couple [Au_2_(dmpm)_2_^3+^]*/[Au_2_(dmpm)_2_^2+^]*.

[Au_2_(dppm)_2_]^2+^ is a photocatalyst for C−C bond formation with the substrates PhCH_2_Cl and CH_3_(CH_2_)_4_Br.^186^ The intimate mechanism most likely includes homolytic bond cleavage and atom transfer [Eq. (3)].

(3) $${}^{3}\left[\mathrm{Au}{}_{2}\left(\mathrm{dppm}\right){}_{2}{}^{2+}\right]{}^{*}+R\text{-}X\to \left[\mathrm{Au}{}_{2}\left(\mathrm{dppm}\right){}_{2}X\right]{}^{2+}+R{}^{•}$$

In apparent contradiction with other results (see below), no emissive exciplex formation, either with quenching halogenated solvents or with CH_3_CN or MeOH (which afford different emission lifetimes for the reactive “intrinsic” triplet state), was observed. In a later article published by the same group, primarily competitive exciplex formation of the activated diphosphine complex with solvent has been held responsible for its inactivity in C−H bond activation.^187^

Parallel to most of the work described above, other di‐ and polynuclear Au–Au‐bonded luminescent complexes were discovered and investigated.^188^, ^189^, ^190^, ^191^ In these systems, ligands were involved in the absorption and emission electron transfer processes; Au−Au covalent bond formation was not an important consideration. Yet, it may be expected that excitation of electrons from antibonding HOMO d orbitals in d^10^–d^10^ systems into whichever bonding orbitals are available could also strengthen the Au⋅⋅⋅Au association to varying degrees. In this Review, we focus mainly on complexes exhibiting compelling MC excitations and emissions. Transitions localized on ligands, or metals and ligands, are not considered in detail except when the metal–metal bond is clearly strengthened (Section 4.2.4). For other developments, the reader is referred to overview articles by the groups of Gade,^192^ Bowmaker,^193^ Fackler,^56^ and Yam.^194^

Despite much research activity, towards the end of the 20th century, no substantial experimental or theoretical evidence for, or information on, Au^I^ −Au^I^ bonding in the excited state of double‐bridged diphosphine complexes of gold had become available. Soon thereafter, important progress with regard to the excited states themselves and the molecular dynamics of the activated complexes was reported by Che and co‐workers, in particular Phillips and Zhang.

#### 4.2.2 Advanced Experimental and Theoretical Investigations

With bis(dicyclohexylphosphino)methane (dcpm) as an optically transparent bridging ligand, spectroscopic studies revealed that complexes of the type [Au_2_(dcpm)_2_]Y_2_ (Y^−^=ClO_4_^−^, PF_6_^−^, CF_3_SO_3_^−^, [Au(CN)_2_]^−^, Cl^−^, I^−^; formula **N**, R=Cy), subsequent to excitation, exhibit one or two triplet→singlet phosphorescence emission bands in the near‐UV and visible regions.^187^, ^195^ The relative band intensities, and whether only one or both bands are observed, depends on various factors such as the chosen counterion, whether measurements are made in solution, glassy solution, or the solid state, and, if in solution, which solvent is used. For example, in CH_3_CN solution, all of the complexes with non‐coordinating counterions exhibit an intense, low‐energy (ca. 500 nm) emission whereas the high‐energy phosphorescence band (ca. 360–370 nm), when present, is weak. Precisely the opposite is true for the same complexes in the crystalline state. With coordinating Cl^−^ and I^−^ counterions, no high‐energy band is observed in solution or in the solid state. The two well separated luminescence bands have been assigned as originating, respectively, from ^3^[Au_2_(dcpm)_2_^2+^]* as the lower‐energy variant of the initially formed ^1^[Au_2_(dcpm)_2_^2+^]* excimer—affording the so‐called intrinsic band—and from solvated exciplexes, [Au_2_(dcpm)_2_(CH_3_CN)_*x*_^2+^]*, and/or exciplexes bearing one or more counterions, [Au_2_(dcpm)_2_(Y)_*n*_^(2−*n*)+^]*. Note that in these dinuclear excimers, the conventional monomer–dimer conversion is not applicable; the dinuclear, noncovalently bonded ground‐state complex serves as the reference congener for the formed excimer. The transition from the initial singlet excited state to triplet excited states of lower energies, as well as the commensurate structural modification, were soon thereafter described in more detail by quantum‐chemical calculations (see below). Exciplex formation of long‐lived ^3^[Au_2_(dcpm)_2_^2+^]* with halides (X^−^=Cl^−^, Br^−^) can be straightforward complexation [Eq. (4)] or may involve photoredox decay [Eq. (5)].

(4) $${}^{3}\left[\mathrm{Au}{}_{2}\left(\mathrm{dcpm}\right){}_{2}{}^{2+}\right]{}^{*}+X{}^{-}\vec{\leftarrow }{}^{3}\left[\mathrm{Au}{}_{2}\left(\mathrm{dcpm}\right){}_{2}X{}^{+}\right]{}^{*}$$

(5) $${}^{3}\left[\mathrm{Au}{}_{2}\left(\mathrm{dcpm}\right){}_{2}{}^{2+}\right]{}^{*}+2\;I{}^{-}\to \mathrm{Au}{}_{2}\left(\mathrm{dcpm}\right){}_{2}{}^{+}+I{}_{2}{}^{-}$$

As mentioned before, exciplex formation between complexes ^3^[Au_2_(P^P)_2_^2+^]* and solvent makes such complexes less accessible for C−H bonds of substrates where the interaction involves an inner‐sphere mechanism.^187^ An investigation of the excited‐state reactivity of Au−Au‐bonded [Au_2_(dpim)_2_]^2+^ complexes (dpim=2‐(diphenylphosphino)‐1‐methylimidazole) confirmed the dependence of the emission energy on the coordination ability of the counterion in accordance with Che's exciplex proposals.^196^

The linear trinuclear complexes [Au_3_(dcpm)_2_]Y_3_ (Y=ClO_4_^−^, PF_6_^−^, CF_3_SO_3_^−^, Cl, SCN^−^) in the solid state and in CH_2_Cl_2_ solution exhibit very much the same relaxation behavior as their dinuclear counterparts described above.^197^ Both the higher‐energy intrinsic peaks and the lower‐energy emissions—the latter here pertinently attributed to exciplex formation with the coordinating counterions Cl^−^ and SCN^−^—are, however, red‐shifted for trinuclear complexes and found in the respective wavelength regions 442–452 and 558–634 nm. DFT calculations support a previous result^163^ that the emission energies in polynuclear linear‐chain complexes show an inverse relationship with the number of metal atoms in the chain. Emission energies below 500 nm in linear polynuclear gold complexes should not be explained in terms of Au−Au exciplex bonding. Notably, no C−X bond rupture of the haloalkane solvent occurred during all of the processes in solution (see below).

Che and co‐workers^152^ reported a Raman spectroscopic study of the compound [Au_2_(dcpm)_2_](ClO_4_)_2_ directed at the lowest energy, dipole‐allowed absorption band at about 277 nm in solution involving the generally accepted ^1^[dσ*pσ] excited state. By using various formalisms and the Condon approximation, it was established that the dσ*→pσ excitation is consistent with the Raman results. The change in the Au−Au bond length from the singlet ground state (or the initial Franck–Condon, FC, product) to the first vibrationally relaxed singlet excited state amounts to 0.11 Å, giving a bond length of 2.81 Å for the double‐bridged excimer. The Au^I^ −Au^I^ σ‐bond, presumably formed by p–p overlap, is somewhat longer than the ca. 2.6 Å distance separating gold atoms in dinuclear Au^0^ and Au^II^ complexes, for which a larger contribution by s‐orbitals in the bonding was invoked.^7^ All further discussion in this section refers only to electronic ground and excited states. Vibrational and rotational energy levels are not included.

Comparative absorption and resonance Raman spectroscopic studies of [M_2_(dcpm)_2_]^2+^ complexes of copper, silver, and gold have confirmed that M−M bonds in all three complexes are strengthened upon MC excitation.^198^, ^199^, ^200^ The change in bond length associated with the singlet–singlet transition follows the order Δ_Cu−Cu_>Δ_Ag−Ag_>Δ_Au−Au_. In the case of gold complexes, solvent complexation in the activated state apparently stimulates Au−Au bond tightening, whereas coordination by a third strong ligand donor as in complexes of the stoichiometry [M_2_(dmpm)_3_]^2+^, where the coordination number has increased from CN=2 to CN=3, effects Au−Au bond extension or even cleavage, both in the ground and excited states.

Two significant theoretical papers on [Au_2_(P^P)_2_]^2+^‐ and [Au_2_(P^S)_2_]^2+^‐type complexes were published in the early 2000s by Che and Zhang^201^ and Zhang and Pan.^202^ The computational model ligands diphosphinomethane (H_2_PCH_2_PH_2_, dpm), *C*‐mercaptophosphinomethane (HSCH_2_PH_2_, mpm), and *C*‐(methylsulfido)phosphinomethane (MeSCH_2_PH_2_, mspm) were employed^201^ to represent neutral, ditopic diphosphine and phosphine–thioether ligands in the respective dinuclear metallacyclic gold complexes, all in the chair formation. Notably, the ligand lone pair electrons populating the gold 6s and 6p orbitals were added to the naked [Au_2_]^2+^ systems, and the formal 5d^10^6s^0^ closed‐shell configuration of each participating gold atom is modified, allowing additional physical bonding forces to become operative. This intervention, combined with instantaneous London dispersion forces, necessitates, as mentioned earlier, the use of configuration interaction methods to correctly describe the aurophilic interaction in the ground state. The authors used an MP2‐based approach to optimize the ground‐state structures. To describe the excited states, the single‐electron configuration interaction method (CIS) was employed, which possibly suffices, in view of the more important role of orbital overlap compared to the ground state. To theoretically mimic the roles of solvent and counterions, respectively, separate calculations were carried out for the model complexes [Au_2_(dpm)_2_(CH_3_CN)_2_]^2+^, [Au_2_(mpm)_2_(CH_3_CN)_2_]^2+^, [Au_2_(mspm)_2_(CH_3_CN)_2_]^2+^, [Au_2_(dpm)_2_](ClO)_2_, and [Au_2_(mpm)_2_](ClO)_2_; the latter two simulate the solid‐state behavior of [Au_2_(P^P)_2_](ClO_4_)_2_‐ and [Au_2_(P^S)_2_](ClO_4_)_2_‐type complexes. The proposals made by Che and co‐workers regarding the presence and position of two prominently emitting triplet states were found to be realistic. Relative term energies for the proposed excited states of the excimer [Au_2_(dcpm)_2_^2+^]* and its exciplexes formed by association with solvent molecules were approximately reproduced by the modelling studies.

**Scheme 8:**
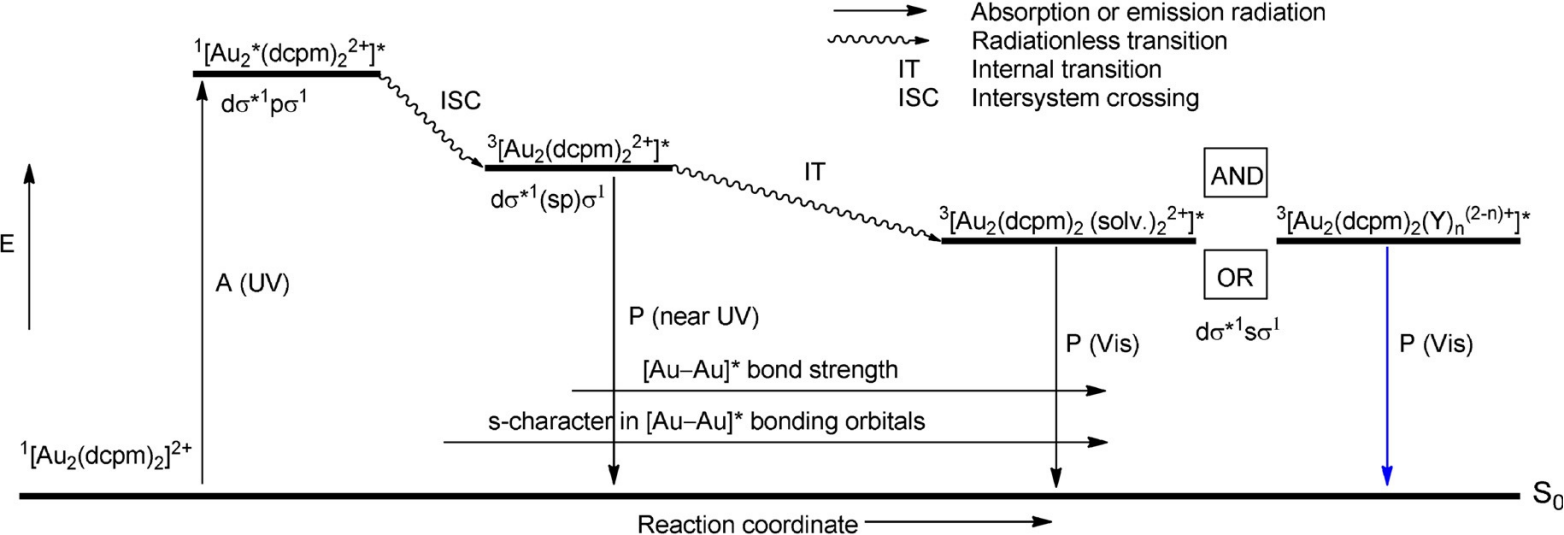
Qualitative, highly idealized Jablonski energy level diagram showing the different species accounting for absorption and emission of [Au_2_(dcpm)_2_]Y_2_ in solution and in the solid state. A=absorption; P=phosphorescence.

The computational results of selected [Au_2_(P^P)_2_]^2+^ complexes have shown that excitation in the UV region is MC, affording a 5dσ*^1^6pσ^1^ electronic configuration. Covalent bond formation in the excited state is initially accomplished by p_*z*_–p_*z*_ atomic orbital overlap. Two lower‐energy, long‐lived triplet states are predicted, the higher of which (the intrinsic state for an unsolvated complex) emits in the near‐UV region and corresponds to a 5dσ*^1^(sp)σ^1^ electron configuration. The stronger Au−Au bond in this instance is formed by overlap of significantly hybridized 6s and 6p atomic orbitals. Finally, linearly combined 6s orbitals are solely involved in the Au−Au bond formation for the lowest‐energy triplet state, ^3^[5dσ*^1^6sσ^1^], explaining the appearance of short Au−Au bonds. This state corresponds to exciplex formation by complex–solvent and/or complex–counterion interactions. Overall, the bond contraction upon electron excitation was calculated to be much more significant than estimated from results of Raman experiments. According to Che and co‐workers,^195^ the unsolvated situation finds its practical counterpart in crystalline, double‐bridged bis(diphosphine) complexes when the counterion is positioned remote from the metal centers. For cases where cation–anion electrostatic interaction gains importance, theoretical modeling shows that the Au−Au interaction strengthens, and the solid‐state emission is red‐shifted, similar to the situation calculated for the assembly when solvent molecules are added to the model complex. Concurrent to the strengthening of the Au−Au interactions by excimer and exciplex formation, the metal–ligand bonds (Au−P and Au−S of phosphine and thiol or thioether donors, respectively) are weakened and elongated. In Scheme 8, the absorption and emission results for [Au_2_(dcpm)_2_]^2+^ are combined with theoretical information pertaining to related model complexes in a Jablonski diagram, showing the trajectory of the [Au−Au]* bond length variation along the reaction coordinate. We mention that in model complexes of the general type [Au_2_(HSCH_2_PH_2_)_2_]^2+^, the lowest‐energy emission has a small MMLCT component.

According to a recent ultrafast, time‐resolved transient absorption (TRTA) and time‐resolved emission (TRE) spectroscopic study,^203^ the intrinsic excimer in the triplet state is formed immediately (i.e., within 0.15 ps) upon irradiation of [Au_2_(dcpm)_2_](ClO_4_)_2_ as a common process that proceeds at an ultrafast rate, with no solvent or phase intervention. The triplet state decays with a lifetime of 4.3 μs to the ground state. When the gold complex is dissolved in CH_3_CN, subsequent fast exciplex formation by sequestering of solvent molecules takes place (time constant 4.5 ps). This process illustrates the tendency of activated Au^I^ complexes to increase their coordination number above 2. With CH_2_Cl_2_ as the solvent, the outcome (not shown in Scheme 2) is completely different, and the inherent triplet state is transformed within 510 ps to a triplet phosphorescent intermediate (of unknown composition) through C−X bond cleavage [compare Eq. (3)]. Timescales and structural information on the dynamics of exciplex formation by counterion binding [Eq. (4)] are still lacking.

Whereas in Au^I^⋅⋅⋅Au^I^‐associated complexes, Au−Au bond shortening is the most important distortion mode in the excited state, Au(PR_3_)_3_‐type complexes are rearranged upon excitation by a symmetry change from trigonal to T‐shaped.^204^ This theoretical result contradicts previous interpretations according to which large Stoke shifts in such three‐coordinate complexes can be attributed to gold–ligand bond contraction.^205^, ^206^

#### 4.2.3 Related Observations in Di‐ and Trinuclear Au^I^ Complexes with Bridging Phosphine‐Carbene Ligands

An unsymmetrical dimetallacyclic complex with carbene/phosphine‐functional ligands has recently been reported by Danopoulos, Braunstein, and co‐workers.^168^ The single most significant feature of their P^C^P ligand in coordination and spectrochemical contexts are the two *exo* phosphine groups, one of which is dangling in **P**, while all three donor groups are coordinatively involved in the linear trinuclear Au_3_ chain found in complex **Q** 
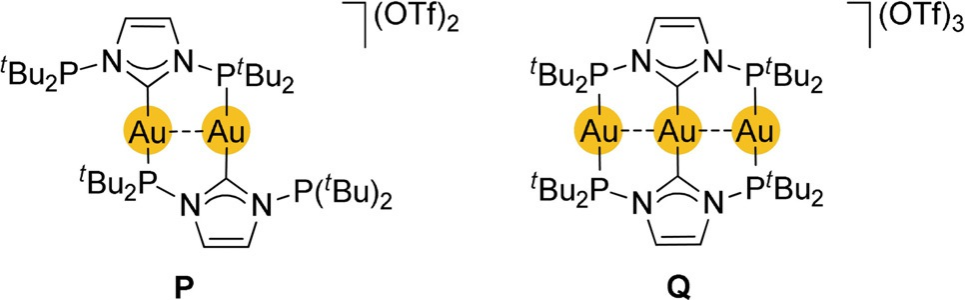
, [Au_3_L_2_](OTf)_3_, which is reminiscent of the [Au_3_(dmpm)_2_]^3+^ chelates investigated by Yam and Wong,^194^ and of a mixed complex salt described by Deák and co‐workers.^207^ Only compound **Q** exhibits a band of an intense, almost pure MC absorption in solution (*λ*_max_=345 nm) in addition to other bands of higher energy with ligand orbital involvement. The important role of the dσ*^1^pσ^1^ configuration and significant Au−Au covalent bonding in the excited state has been demonstrated by resonance‐enhanced Raman spectroscopy following the MC transitions, showing the excited‐state vibrations of the Au−Au bonds. Here we mainly concentrate on the results for **Q**. This compound displays a narrow, intense, blue‐violet radiation band at 446 nm, essentially independent of solvent polarity, state (solution, glassy matrix, neat powder), and temperature, and with the nature of an MC ^3^[5dσ*6pσ] triplet state.^197^ The results of chemical computations (DFT level) have indicated that for **P** in the excited state, coordination of the dangling phosphine group may contribute to the stabilization of the emitting triplet state. The shortening of the Au–Au distances in the excited state of compound **Q** seems unambiguous, despite the use of rigid ligands. Quantitative comparison between ground‐ and excited‐state distances is not really meaningful in view of the inadequacy of DFT calculations for the ground state.^7^, ^208^ Notably, the Au−P and Au−C distances show only very minor changes upon excitation.

#### 4.2.4 Heteroatom Involvement in Au^I^–Au^I^ Bonding

Luminescent, binuclear, phosphine‐bridged gold(I) complexes that also contain thiolate ligands (single‐bridged, generic form **R**; double‐bridged, complex form **S** 
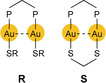
) as well as simpler, mononuclear, R_3_PAuSR′‐type complexes have been studied experimentally by various research groups.^209^, ^210^, ^211^, ^212^, ^213^ In all of these examples, the S‐donor lone pair electrons (designated S(pπ)) are intimately implicated in the excitation and emission processes and, ultimately, in [Au−Au]* bond formation. Under favorable, idealized conditions for Au−Au bonding, the two gold atoms are positioned in close proximity, and the initial lowest‐energy excitation is qualitatively described as S(pπ)→pσ electron transfer (or ligand to metal metal charge transfer, LMMCT),^212^, ^213^ where pσ represents the bonding LUMO formed by overlap of localized p_*z*_ orbitals in the Au_2_ unit.^209^, ^211^, ^212^ No structural information about the relaxation dynamics of the resulting ^1^[LMMCT]* and ^3^[LMMCT]* states and hence about progressive excimer or exciplex formation is available.

Importantly, structural studies in combination with luminescence spectroscopy have shown that in various phosphine‐thiolate complexes of Au^I^, the strength of the ground‐state Au⋅⋅⋅Au aurophilic interaction is not necessarily the dominant factor in determining the solid‐state emission energetics; nor is it always directly correlated with such energies. The composition and spatial orientation of the thiol ligands, for example, could be equally important or even play a decisive role.^210^, ^211^ Zhang and Pan^202^, ^214^ have calculated the orbital involvement during optical transitions in three neutral model complexes, [Au_2_(dpm)(SCH_2_S)], [Au_2_(H_2_PCH_2_S)_2_], and [Au_2_(HSCH_2_S)_2_], by using MP2 and CIS methods. Their results confirmed the interpretation given above regarding Au−Au bond formation and strengthening in the excited state subsequent to S(pπ)→sσ and/or pσ electron promotion. Although the assignment of the lowest‐energy emissions in the three complexes is less straightforward, they are all assumed to originate from triplet metal‐metal localized states.

The mode according to which soft heteroatom‐donor ligands contribute to covalent Au−Au bond formation at the time of UV/Vis electron excitation has been placed on a firmer footing by the results of Saillard, Liu, Boucekkine, and co‐workers.^215^, ^216^ The dinuclear, diseleno‐ and dithiophosphinate complexes **T** and **U** 
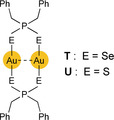
 were investigated by experimental (single‐crystal X‐ray diffraction; excitation, emission, and Raman spectroscopy) and computational (time‐dependent DFT) means.

Very weak Au⋅⋅⋅Au interactions at intramolecular distances of 3.387 and 3.456 Å for **T** and 3.267 Å for **U** are characteristic of these complexes. The structureless, lowest‐energy excitation and emission bands exhibit their low‐temperature (77 K) maxima, *λ*_max_^exc^ and *λ*_max_^em^, in solid 2‐MeTHF at 434 and 593 nm for **T**, and at 471 and 565 nm for **U**. Solution spectra are all hypsochromically shifted. To a first approximation the excitation and emission processes can again be attributed to single‐electron transfer between the HOMO and LUMO. The former orbitals, *E*(pπ), are constructed by linear combinations of lone‐pair‐containing atomic orbitals at Se or S but also have 12–16 % Au d‐orbital character. The LUMO for **T** carries a dominant contribution from gold p orbitals whereas the LUMO of **U** is composed of 35 % gold (d orbital) complemented by 46 % sulfur character.

During excitation, electron density is transferred from a non‐bonding to a bonding MO localized between two gold atoms; the descriptive σ p–p overlap is responsible for Au−Au bond formation (bond order≤0.5). Results of resonance Raman spectroscopy investigations have clearly confirmed Au−Au stretching motions in the resulting excited singlet and triplet states, which are completely absent in the ground states. According to DFT computations, the distance between the two gold atoms in the triplet excited state contracts by up to 25 % compared to the ground state. These results provide convincing evidence that not only Au⋅⋅⋅Au aurophilic interactions impact positively on Au^I^−Au^I^ bond formation but that soft heteroatom‐donor ligands may also be important electron sources for such binding. On the other hand, the proposed LMMCT designation for the excitation processes perhaps underestimates the significant involvement of heteroatom orbitals in the LUMO acceptor orbitals. Rehybridization along the reaction coordinate is predicted by NBO analysis.

### 4.3 Di‐ and Polynuclear Excimers Arising from Mononuclear Au^I^ Complexes

#### 4.3.1 The Dicyanoaurate(I) Case

Compounds containing the [Au(CN)_2_]^−^ anion serve as prime and prototype examples of linear mononuclear Au^I^ complexes participating in intermolecular excimer and exciplex formation upon excitation. Several early spectrophotometric investigations have shown that solutions of these compounds exhibit photoluminescence in concentrations ≥0.01 m. This result has been attributed to oligomerization in solution, a process ensuing from cooperative aurophilic interactions (see above).^101^

In an important and directional paper, Patterson, Fackler, and co‐workers^107^ described the results of an investigation into this luminescence phenomenon by concentrating on excited‐state interactions in solution. Although generally, only two major unstructured emission bands with large Stokes shifts have been observed in aqueous solution at ambient temperature, a multitude of emission bands may be generated and separated from one another by varying the concentration, solvent, temperature, and excitation wavelength. In general, weakly Au⋅⋅⋅Au‐associated dimers or oligomers form the corresponding excimers upon selective excitation; related, higher‐order exciplexes are realized when such excimers interact with and bind to additional ground‐state monomeric complex units. Beginning with the highest‐energy emission, which has been ascribed to a triplet state of the lowest‐nuclearity excimer [Au(CN)_2_^−^]_n_* (i.e., *n*=2), the progressively lower energy bands may be attributed to growing oligomers (*n*>2; exciplexes) formed by the successive addition of monomers to the oligomer chain (formulae **I, J** above).

In the focus of the investigation were the changes in Au−Au bond length and strength that parallel the spectroscopic transitions from higher‐ to lower‐energy emission bands. It should be noted that exciplex formation by engagement with solvent, as earlier proposed for double‐bridged digold phosphine complexes in the excited state, has been ruled out because the solution‐ and solid‐state emission bands are similar in (initial) shape and energy.

Some of the assumptions and proposals mentioned above have been scrutinized in the same paper by MP2 chemical computation using [AuCN)_2_^−^]_2_ in the staggered dimer conformation as a model compound (formula **I** above). Upon excitation and excimer formation, the calculated Au−Au distance decreases from 2.96 Å to 2.66 Å. Simultaneously, the calculated Raman active stretching frequency, ν_Au−Au_, almost doubles, and the force constant increases by one order of magnitude. The covalent Au−Au bond in the excited state is, again, not much longer than in well‐known unsupported Au^II^−Au^II^ complexes linked by gold–gold single bonds (ca. 2.6 Å, above). Extended Hückel excited‐state calculations distinctly show that the [Au^I^ −Au^I^]* bonds increase in strength with increasing oligomerization along the series [Au(CN)_2_^−^]_*n*_: the exciplex [Au(CN)_2_^−^]_3_*, for example, is more strongly bonded (lower triplet energy) than the excimer [Au(CN)_2_^−^]_2_*. In addition, the Au−Au bonding is influenced by the geometry (linear, bent) and conformation (eclipsed, staggered) of a particular exciplex. These aspects are addressed in greater detail below. Interestingly, although the [Ag(CN)_2_]^−^ complex photochemically generally mimics its heavier analogue, the Au⋅⋅⋅Au aurophilic interactions are stronger than the Ag⋅⋅⋅Ag argentophilic interactions in the ground state while in the corresponding excited state, the Ag−Ag interaction is stronger than the Au−Au interaction.

The first study of luminescence in pure single crystals of K[Au(CN)_2_] was undertaken in Patterson's laboratory in 1986.^95^ Even though the investigations and rationalizations focused on variations in ground state Au–Au atomic overlap, the results of this investigation formed the basis of the tuning approach that was developed later. Excimer and exciplex tuning comprises the selective generation of separated emission bands for a chosen luminescent system by variation of various experimental parameters explained below. Tuning research has received much attention in recent years due to potential optochemical applications in excitomic energy transfer,^217^ which could lead to photocatalysis—even in bulk processes, such as the decomposition of pesticides for decontamination of industrial effluents.^218^ Significant tuning has been achieved for [Au(CN)_2_^−^]_n_* present in pure K[Au(CN)_2_] and K[Au(CN)_2_]/KCl doped crystals.^103^

Association of the anions in crystalline K[Au(CN)_2_] and Cs[Au(CN)_2_] leads to the formation of sheets of gold atoms (Figure 1).^98^ To a first approximation, the electronic situation within the crystals is described in terms of electron configurations in one‐electron energy bands. The valence band (VB) is mainly composed of the 5d${}_{z{}^{2}}$ and 6s orbitals of the participating Au atoms, and the conduction band (CB) comprises essentially 6p_*z*_ orbitals, mixed with small contributions of ligand orbitals. Spectroscopic transitions are then simply described as electron transport from VB to CB or vice versa. A more complete description of spectroscopic states requires, amongst other aspects, a consideration of electron–electron (term generation), hole–electron (delocalized exciton formation), and exciton–lattice (effecting localized, self‐trapped states) interactions. The second‐order exciton–lattice interaction leads to a reduction in Au−Au distance and stronger bonding within the chains or sheets of gold atoms. A detailed theoretical description is not yet available for the [Au(CN)_2_]^−^ layers in M[Au(CN)_2_] crystals, but has been applied to quasi one‐dimensional M_*n*_[Pt(CN)_4_]⋅*x* H_2_O compounds (M=Na, K, Ba, Mg, etc.; *n*=2 or 1).^219^

Single, broad emission peaks (at 397 and 435 nm, respectively) have been recorded for K[Au(CN)_2_] and Cs[Au(CN)_2_] at room temperature.^98^ With hydrostatic pressure applied (up to 40 kbar), large, linearly related red‐shifts of, respectively, −300 cm^−1^ kbar^−1^ and −200 cm^−1^ kbar^−1^, are observed. The shifts are related to the relative crystal compressibility. At a microlevel, the effect of increased pressure stems from shrinking Au−Au bonds in the excited state compared to the ground state; the band gap closes and the band energy is lowered. The broad peak shown by Cs[Au(CN)_2_] is resolvable at 5 K into two dominant emission peaks. The one at lower energy (454 nm) has the properties of a localized state most likely involving a cluster of only a few [Au(CN)_2_]^−^ complex units in analogy to findings for M_*n*_[Pt(CN)_4_] examples. The one at higher energy corresponds to the delocalized cluster states {[Au(CN)_2_]_*n*_}^n−^. The relationship of the emission energies with the Au−Au distances in the crystals is comparable to effects reported for the exciton–lattice interaction in, for example, the one‐dimensional arrays of complex anions in crystals of Ba[Pt(CN)_4_]⋅4 H_2_O.^220^

An increased dopant (K[Au(CN)_2_]) concentration within KCl crystals affords higher oligomers and stronger Au−Au bonding in the excited state, resulting in red‐shifted emission bands.^103^ Different structural versions of a given oligomer also affect the strength of Au⋅⋅M.>Au interactions, as has been shown by Schmidbaur in a different context.^221^ The contraction effect is larger in the activated state than in the ground state. Furthermore, by varying the excitation wavelength for a particular crystal, an increasing number of emission sites, corresponding to long‐lived excited states of at least three exciplexes, may be produced.

The numbers and frequencies of Raman bands observed for crystalline [Au(CN)_2_]^−^‐doped materials strongly correlate with the photoluminescence bands of the different crystals obtained from sample batches of varying dopant concentrations. This result indicates that in the crystal, the [Au(CN)_2_^−^]_*n*_ ions are found in different environments, depending on the value of *n*. Each of the ground‐state oligomers and the corresponding exciplexes that are formed upon excitation experience Au−Au single σ bond formation, which manifests itself in a particular emission band from a triplet excited state. It should be kept in mind that two or more emission bands may overlap when more than one exciplex with a rather similar binding energy is present.

Expanded exciplex tuning (22 000 cm^−1^ range) with selective variations in Au−Au bonding is achievable upon varying the medium (pure crystals, doped crystals, solutions), the temperature, the complex concentration, the counterion, and the host crystal employed.^106^ Figure 9 contained in both Refs. 103 and 107 highlights the amazing success that has already been achieved. In the context of this Review, tuning not only refers to the successful separation of emission bands in order of decreasing energy, but simultaneously affords an excimer and exciplex energy profile showing the Au^I^ −Au^I^ bond strengthening along the reaction coordinate.

Crystals or powders of the compound (DNP)[Au(CN)_2_]_2_⋅4 H_2_O (DNP^2+^=1,1′‐bis(2,4‐dinitrophenyl‐4,4′‐bipyridinium)), where the anions are arranged in dimers between the large cations, exhibit two emission bands in the visible region at 10 K, which are attributable to the DMP^2+^ and {[Au(CN)_2_]_2_}^2−^ units.^116^ Only the latter band is of interest here. The near‐UV excitation and emission at about 420 and 580 nm, respectively, are standard for [Au(CN)_2_]^−^ salts. At temperatures above 50 K, owing to the presence of the DNP^2+^ cations, rapid quenching by means of electron transfer occurs, similar to the consequences of the reaction between ^3^[Au_2_(dppm)_2_^2+^]* and MV^2+^ [Eq. (2)].^163^, ^183^

Five recent papers not only support almost all of the results obtained in Patterson's laboratory regarding the origin and assignment of emission bands for more concentrated [Au(CN)_2_]^−^ complex solutions and the type of excimer/exciplex formation responsible for luminescence properties, but they also cast much new light on the ultrafast structural dynamics of such activated chemical processes subsequent to initial excitation.

The ultrafast dynamics of Au−Au bond formation of the staggered dimer [Au(CN)_2_^−^]_2_ in the excited state has been clarified by femtosecond and picosecond time‐resolved transient absorption (TRTA) spectroscopy. Predominantly dimers are present in very dilute [Au(CN)_2_]^−^ solutions (ca. 0.04 m) and are the only species undergoing excitation in the near‐UV region (ca. 270 nm). Relaxation transitions as well as the time constants for subsequent processes have been determined.^109^

**Scheme 9:**
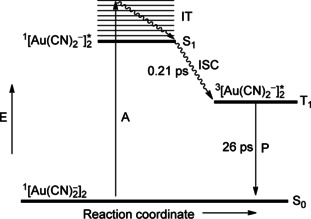
Simplified Jablonski diagram for the excitation and emission of the dimer [Au(CN)_2_^−^]_2._ A=absorption; IT=internal transition; ISC=intersystem crossing; P=phosphorescence.

Upon photoexcitation (Scheme 9), coherent molecular motions of the excited dimer in the S_1_ singlet state present in the femtosecond time region reflect the rapid formation of a rigid covalent Au−Au bond. Further Au−Au bond contraction occurs during subsequent intersystem crossing into a transient, low‐energy triplet state of the dimer T_1_ (time constant 0.21 ps). This T_1_ steady state slowly decays (emission at 330 nm) with a time constant of 26 ps. No further oligomerization in the excited state occurs under the selected experimental conditions.

Electron structure calculations for the oligomers [Au(CN)_2_^−^]_*n*_ (*n*=2–5) have been carried out by Dolg, Thiel, and co‐workers at the MP2 level (ground state; first excited state of the dimer), and at the DFT, RI‐CC2, and MSCASPT2 levels of theory (excited states).^108^ The theoretical results for the dimer are in full agreement with those presented by Iwamura, Tahara, and co‐workers^110^ for the staggered structure. In contrast to the ground state, where the eclipsed isomer is unstable, the excited‐state bonding configurations dσ*^1^pσ^1^ and dσ*^1^pπ^1^ of the eclipsed dimer have very similar energies in both the S_1_ and T_1_ states, and may theoretically also contribute to the absorption and emission bands. The calculated Au−Au bond lengths for the two conformations in both the activated singlet and triplet states do not differ significantly; they are both about 0.3 Å shorter than in the corresponding ground states. As the experimental results for the dimeric complex are in agreement with the proposed structural dynamics of the staggered isomer, the rather complex spectroscopic transitions calculated for the eclipsed form are not discussed further.

Following in the footsteps of Patterson, Fackler, and co‐workers, and relying on the latest applicable experimental and theoretical characterization approaches, other research groups have delved deeper into the solution photophysics and photochemistry of [Au(CN)_2_^−^]_*n*_ oligomers, particularly those of the trinuclear complex (*n*=3). Iwamura, Tahara, and co‐workers^109^ have used TRTA spectroscopy as well as time‐dependent DFT calculations to explain structural changes during photochemical excited‐state emission transitions. After they had carefully separated the excitation and emission transitions of the trimer [Au(CN)_2_^−^]_3_ from other interfering photochemical transitions, it was concluded that at least five different kinetic events and five complexes are required to fully explain the absorption and emission bands. Three time constants for successive transitions were determined. A decisive and coherent oscillatory motion identified in the femtosecond region has provided strong evidence for ultrafast Au−Au bond formation in the activated state.

**Scheme 10:**
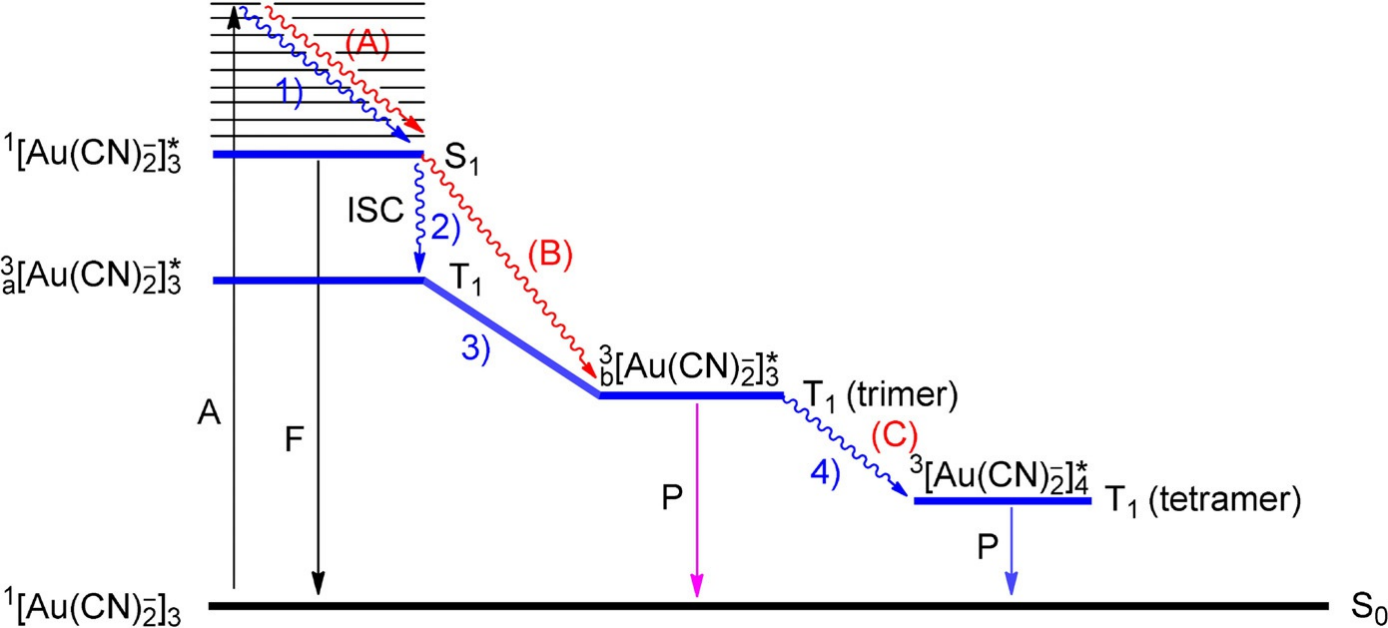
Jablonski diagram for the spectroscopic energy levels of the trimer [Au(CN)_2_^−^]_3_ 109 (in blue); structural changes: 1) Au–Au bond shortening, 2) ISC within 500 fs of excitation, 3) bent‐to‐linear structural modification in the triplet state within 2.1 ps, 4) association of ^3^_b_[Au(CN)_2_^−^]_3_* (linear, lifetime 2 ns) with a ground‐state monomer to form a linear tetramer. (A)–(C) (in red, where different) indicate the structural changes later proposed in Refs. 108 and 111, 112, 113.

All of the proposed spectroscopic transitions with their time constants and the concomitant structural changes are illustrated in Schemes 10 and S1 (see the Supporting Information). Note that the weakly associated trimer [Au(CN)_2_^−^]_3_ present in solutions of a given concentration under ambient conditions features a bent (non‐linear) ground‐state structure that remains unchanged in the Franck–Condon region upon excitation but relaxes immediately into a similar but covalently bonded structure. The Au−Au bond tightening is apparently completed during this vibrational relaxation (step 1). The singlet excited state S_1_ is fluorescent. Intersystem crossing (step 2) to form ^3^_a_[Au(CN)_2_^−^]_3_* is complete within 500 fs. The measurable second kinetic component (step 3) comprises simply the unfolding of the Au_3_ frame into a staggered linear arrangement in ^3^_b_[Au(CN)_2_^−^]*, which is then followed by oligomer growth whilst maintaining the linear arrangement of the gold atoms (step 4). The lowest energy triplet state loses energy by phosphorescence. Transient energy changes based on emissive bands and numerical time constraints have been experimentally determined, but the assignment of diverse structural changes rests on hypothesis and calculation.

The emission wavelengths (energies) and lifetimes of [Au(CN)_2_]^−^ oligomers in aqueous solution are influenced by the molecular environment in the excited state. Surprisingly, the [Au(CN)_2_^−^]_n_* (*n*≥4) oligomers decay by dissociation into smaller excimers or exciplexes but interactions with tetraalkylammonium cations such as Et_4_N^+^ were found to stabilize the larger oligomers and enhance the lower‐energy emissions.^222^

Photoexcitation of dicyanoaurate anions in ionic liquids of 1‐butyl‐3‐methylimidazolium and *N*‐butyl‐*N*‐methyl pyrrolidinium cations in the near‐UV region produced one fluorescent band at approximately 380 nm and phosphorescence at 460 nm. The peak position of the latter band was shifted to lower energy over time. Two time constants were determined for each ionic liquid, corresponding to triplet formation and relaxation, respectively. The authors of this paper^114^ referred to the dynamics proposed by Iwamura and co‐workers for the trimeric species [Au(CN)_2_^−^]_3_* (Scheme 10) to explain their data. Two structural situations of the triplet trimers were again invoked even though experimental evidence for only one was available. The peak shift towards longer wavelengths was interpreted as being due to the formation of higher [Au(CN)_2_^−^]_n_* oligomers influenced by the size of the cation.

The interpretation of progressive changes occurring in the activated state upon excitation of [Au(CN)_2_]^−^ solutions by Iwamura, Tahara, and co‐workers has been challenged in more recent articles. Therein, the authors agreed in general with the proposed features but the analyses differed in terms of the detailed kinetics of the early transitions.

Working at the same level of theory that had been used for interpreting the photophysics of the [Au(CN)_2_]^−^ dimer (see above), Fang, Dolg, Thiel, and co‐workers^108^ came to the conclusion that the staggered bent structure for the trimer does not exist in the vibrational ground states of the singlet or triplet excited states. Hence, the linear arrangement is already rapidly adopted during vibrational relaxation in the activated singlet state and accompanied by Au−Au−Au bond shortening ((A) in Scheme 10). Subsequently, intersystem crossing ((B) in Scheme 10) affords a low‐energy triplet for the trimer, which corresponds only to the final linear structures in Schemes 10 and S1.

Motivated by the contrasting results described in the previous paragraph, Kim and co‐workers^111^, ^112^ embarked on the first time‐resolved X‐ray solution scattering (TRXSS) investigation directed at dissolved gold complexes. They focused explicitly on the photoinduced dynamics of [Au(CN)_2_]^−^ complex systems upon, and subsequent to, excitation by an X‐ray free‐electron laser, which allows ultrafast reactions to be explored. The great advantage of TRXSS over TRTA is its sensitivity towards the global molecular structure in solution. The technique is therefore well suited to directly characterize the structures of individual dominant complex transient species with sub‐Å spatial resolution. With time limits also determinable at the sub‐ps level, information on bond contraction, covalent bond formation, bent‐to‐linear transformation, and oligomer growth can be provided along a chosen reaction coordinate.

The results reported by Kim and co‐workers are in full agreement with those obtained earlier by chemical computation.^108^ In contradiction to the findings of Iwamura, Tahara, and co‐workers, the following relaxation pathway was established (compare Schemes 10 and S1): A) Excitation immediately (within 500 fs) causes the bent‐to‐linear transformation in the Franck–Condon region; B) the single involved triplet situation is reached by S_1_→T_1_ (trimer) ISC within 1.6 ps before intermolecular interaction occurs, and comprises a further tightening of the Au−Au−Au bonds; C) the kinetic time constant for the spectroscopic T_1_(trimer)→T_1_(tetramer) transition by Au–Au atomic orbital overlap is 3 ns; and D) 100 ns are then available for total relaxation to the ground state, T_1_(tetramer)→S_0_.

Prompted by the objection of Iwamura, Tahara, and co‐workers,^110^ inferring that the discrepancies between the TRTA and TRSXX results could be due to the fact that Kim and co‐workers had not used the optimal wavelength for selective trimer excitation, the latter group repeated all of the measurements using two different solutions while exciting at lower energy (now 310 nm vs. 287 nm previously). Compellingly, exactly the same conclusions had to be drawn as before. In the words of Kim and co‐workers, “*the bent‐to‐linear transition of the [Au(CN)_2_^−^]_3_ trimer occurs within 500 fs*”, and “*the mechanism of Au−Au bond formation in the [Au(CN)_2_^−^]_3_ trimer is independent of excitation wavelength*”.^113^ It should be mentioned that the experimental Au−Au bond shortening during ISC amounts to only 0.11 Å, with a rather large standard deviation of about 0.04 Å.

In their most recent contribution, Tahara and co‐workers^223^ have supplemented their TRTA results with time‐domain Raman tracking of the ultrafast structural dynamics of [Au(CN)_2_^−^]_3_ upon Au−Au bond formation. The Au−Au breathing vibration has been measured at approximately 90 cm^−1^. The initial rapid growth in resonance Raman enhancement corresponding to Au−Au bond formation and intersystem crossing is followed by a gradual frequency upshift over a few picoseconds supporting a continuous structural change on the triplet potential energy surface such as the proposed bent‐to‐linear transition. Tahara and co‐workers have also discovered that even under almost ideal conditions for trimer excitation, time‐domain Raman measurements are complicated because the presence of an excited tetramer might have an impact on the contrasting X‐ray solution scattering results described above.^108^, ^111^, ^112^

#### 4.3.2 The Di(thiocyanato)aurate(I) Case

The groundbreaking results of Patterson, Fackler, and co‐workers on excimer and exciplex formation of [Au(CN_2_)]^−^ complexes in solution and in doped crystals have served as inspiration for investigations into the luminescence of M[Au(SCN)_2_] complexes in the solid state and in frozen solutions.^140^, ^142^ Uniquely, both blue fluorescence and green, unstructured phosphorescence bands are observed at ambient temperature and at 77 K for the counterion series M^+^=K^+^, Rb^+^, ^*n*^Bu_4_N^+^, and Cs^+^. The major emission band is red‐shifted in frozen solutions compared to crystalline material. A shift to lower energies of the same band is also found with increasing size of the selected counterion. Notably, Ph_4_P^+^ salts, in which the [Au(SCN)_2_]^−^ units are isolated from one another in the solid state, exhibit no gold‐centered emissions. A similar result was previously reported for the cyanide counterpart, ^*n*^Bu_4_N[Au(CN)_2_].^107^

The high‐energy emission corresponds to an emitting excited singlet state (<500 nm), and the other band (>500 nm) arises from a T→S_0_ transition. The molecular structures of the luminescent states and the ground state have been calculated by quantum‐chemical methods at the MP2 and DFT levels, using the dimer [Au(SCN)_2_^−^]_2_ as the model system (formula **K** above). This dimeric unit is realistic as it is present in crystalline ^*n*^Bu_4_N[Au(SCN)_2_]. Excimeric Au−Au bonding in the excited state is predicted to occur concomitant with a significant decrease in bond length of 0.2–0.3 Å (depending on the level of theory employed). On the basis of calculations for aggregates in the gas phase that show a too large red‐shift when the dimer is oligomerized further, the authors hypothesized that discrete dinuclear excimeric units are mainly responsible for the observed luminescence. Such excited dimers, [Au_2_(SCN)_4_^2−^]*, are then present in crystals of the various salts, independent of whether the anions are arranged as dimers, trimers, or chains in the ground state of extended structures in these solids. An influence of cation size on the spectra has been noted. The involvement of counterions is related to unspecified distortions and not to exciplex formation because the larger cations are more influential regarding their bathochromic effect. Most likely, the larger the counterion, the fewer constraints are placed on the photoinduced structural change in the excited state. Frozen solutions allow more freedom than solids.

A “dimer hypothesis” has been contradicted in another study involving neutral complexes of the generic type [(RNC)AuX]. The excited‐state distortion increases and the emission energy decreases when the Au−Au bond shortening “*occurs over a longer range than that in the dimer*”.^224^ Balch and co‐workers,^225^ investigating neutral luminescent (cyclohexylisocyanide)gold(I) halides exhibiting relatively weak aurophilic interactions in the solid state, have also concluded that “*the spectroscopic features […] are a consequence of the supramolecular organization of molecules within the solids*”. Exciplex formation and structural distortion, nevertheless, remain essential features of the excited‐state dynamics in all of these complexes (see Section 4.3.4 below).

#### 4.3.3 Mono‐ and Bis(phosphine)gold(I) Cases

The low‐energy luminescence (*λ*_max_>500 nm) at 70 K of crystalline [(Et_3_P)AuCl] has been attributed to gold‐centered transitions. DFT and ZINDO calculations support this hypothesis. The luminescence is completely quenched in solution.^226^ Structural features of the complexes in the excited states are not known.

The emission spectra of the homoleptic, aurophilically associated cationic complexes [(Me_3_NBH_2_MH_2_)_2_Au]AuCl_4_ (M=P, As) have been studied in the solid state.^146^ The complexes form chains in the crystals, and the two emissions per complex in the blue to violet region have been found (by DFT calculations) to be MC, and to occur between conduction and valence band configurations. The energy of the T_1_→S_0_ intersystem crossing (final relaxation) may be manipulated: Decreasing the temperature manifests itself in a shortening of the ground state Au–Au separation; the orbital overlap in the excited state is enhanced, the band gap decreases, and a red‐shift ensues. The effect of temperature on the Au–Au distances in linear chains of gold complexes in solids to a certain extent mirrors that of increasing hydrostatic pressure as discussed above (Section 4.3.1).

Regardless of the remarkable progress that has been made in understanding the photophysics and chemical transformations of excimers and exciplexes of mononuclear gold(I) complexes preorganized by aurophilic contacts, a number of issues are still unsettled and deserve further attention. These include the role of the cation size and charge in determining the structure and energy of the excited states and their lifetimes in solids and ionic liquids, the excimer characteristics arising in specific positions of a solid matrix and in one‐ or two‐dimensional arrays of gold(I) centers, and details of the relaxation profiles of [Au(CN)_2_^−^]_n_* and [Au(SCN)_2_^−^]_n_* (*n*≥4) in solution.

## 5 The Role of Dinuclear Gold(I) Excimers and Exciplexes in Organic Synthesis

The pioneering investigations of Che and co‐workers into the use of photo‐activated, phosphine‐bridged, dinuclear gold(I) complexes (**N**) as oxidants or reductants, and finally as catalysts, for C−C bond formation^186^ have lately served as an impetus for other research groups to employ such weakly Au⋅⋅⋅Au‐bonded complexes as precatalytic mediators in photocatalysis. The term “photoredox binuclear gold catalysis” now covers a wide range of applications in preparative organic chemistry. Major contributions were independently made by the research groups of Barriault, Hashmi, and others. The most important results have been concisely reviewed by Barriault and co‐workers,^227^, ^228^ and are not repeated in detail here. A variety of reactions including C−X bond cleavage, C−C and C−N cross‐coupling, pinacol rearrangements, and even Heck‐like additions were successfully catalyzed. The [Au^I^ −Au^I^]* excimer fragment existing in a long‐lived triplet state appears as an active center upon irradiation of the reaction mixture with tunable light sources as proposed in the corresponding catalytic cycles. Applications in total synthesis ((±)‐triptolide in Barriault's group;^229^ pyrroloazocine indole alkaloids by Echavarren and co‐workers^230^) and radical polymerization by Nzulu and co‐workers^231^ have convincingly illustrated the power and scope of the approach. A very promising variant using co‐reductants such as tertiary phosphines or thiols was developed by Hashmi and co‐workers.^232^ This and another more recent report^233^ contain key references that allow to follow the current activities.

## 6 Summary and Conclusions

Over the last 30 years, gold chemistry has been identified as a research field where for a plethora of compounds the formation and relaxation of excimers and exciplexes can readily be observed both in the solid state and in solution. This abundance is due the spontaneous aggregation of linear, two‐coordinated gold(I) complexes through side‐on aurophilic interactions. In these aggregates, the gold atoms are held in close contacts with Au–Au distances of approximately 3.0 Å. Upon UV/Vis excitation, these Au⋅⋅⋅Au contacts become significantly shortened, indicating the formation of covalent Au−Au bonds in the excited state. Both the bond lengths and bond energies found and calculated for this novel [Au−Au]* bonding approach those of Au−Au bonding in the gold molecule Au_2_ and its complexes L−Au−Au−L, and also in dinuclear gold(II) complexes.

In typical examples, the UV/Vis excitation is a metal‐centered process [Au_2_]→[Au_2_]*, which leads to a series of singlet and triplet excited states without direct involvement of the ligands. The relaxation is generally manifested qualitatively by luminescence effects, which make the process immediately obvious and allow for detailed quantitative investigations by time‐dependent experiments. Excitation is associated with significant structural changes regarding the bonding of the metal atoms. By contrast, when the excitation involves electronic states of the ligands, that is, when the process is not strictly metal‐centered, comparable effects on the Au–Au bonding are usually less pronounced or absent.

Particularly detailed information on the energy profiles of excimers and exciplexes has been compiled for the dicyanoaurate(I) complex [Au(CN)_2_]^−^, which is associated by aurophilic contacts both in the solid state and in solution. Depending on the concentration in solution or on the doping concentration in solids, dimers, trimers, and tetramers can be observed, and their transformations into excimers and exciplexes have been followed by picosecond/femtosecond time‐resolved emission and absorption spectroscopy and femtosecond solution X‐ray scattering. Resonance Raman spectroscopy has been used as an additional spectroscopic tool to characterize the Au−Au bonding in the [(NC)_2_Au−Au(CN)_2_^2−^]* excimer.

The experimental work has been complemented by extensive quantum‐chemical calculations, which have given deeper insight into the structural characteristics and photo‐induced dynamics of the excited states. Studies of the di(thiocyanato)gold(I) complex [Au(SCN)_2_]^−^ have given similar results, indicating that the characteristics are very general for small gold(I) complexes.

The second area where gold(I) excimers have been found to be very common are dinuclear gold(I) complexes with a cyclic structure in which the intramolecular aurophilic contact is supported by bridging ligands. Upon UV/Vis excitation, the transannular Au⋅⋅⋅Au contact in, for example, an eight‐membered dimetallacyclic compound is shortened to produce a bicyclic structure where the newly generated Au−Au bond is shared by two five‐membered rings. These excimers have again been characterized by UV/Vis absorption and emission spectroscopy, Raman investigations, and finally electrochemical studies estimating the changes in the redox potentials. Interference by the nature of the bridging ligands, counterions, and solvents is more common for the dimetallacycles as they can be investigated in a variety of solvents that can participate in the excitation, emission, and exciplex formation processes. For complexes with typical bis(phosphine) ligands R_2_PCH_2_PR_2_, a recent ultrafast, time‐resolved transient absorption and emission spectroscopic study enabled the identification of the intrinsic excimer in the triplet state (with an Au−Au bond) formed immediately upon irradiation as the product of an ultrafast process, with no solvent or phase intervention. Subsequent interactions may then produce exciplexes with other monomers, solvent molecules, or counterions. Interestingly, the gold atoms in the exciplexes exhibit a stronger nucleophilic character and more readily accept donor molecules or anions than those in the ground state, where the metal centers are strictly two‐coordinate. Cyclic [Au_2_(P^P)_2_]^2+^ complexes produce excimers, which are key intermediates in photoredox binuclear gold catalysis for organic synthesis.

The conclusions drawn from the data accumulated in experimental work have also been confirmed in quantum‐chemical calculations, which have provided estimated characteristics of short‐lived intermediates and transition states. In polynuclear compounds with gold atoms ordered in chains by multifunctional ligands offering different donor atoms, the photophysical performance of their excitons can be tuned by the choice of the ligand characteristics.

Selected examples of silver(I) analogues have also been probed regarding the formation of excimers, and for almost all of them, similar excitation processes have been confirmed. Even though argentophilic interactions in the precursor aggregates are generally considered to be weaker than in the gold analogues, the Ag−Ag binding in the excimers was found to be even stronger, probably owing to the preference of silver(I) for higher coordination numbers, which supports the bonding in exciplexes with three‐ or four‐coordinate metal atoms. This observation shows that strong relativistic effects support more strongly the metallophilic bonding of gold(I) than of silver(I) in the ground state, but are less important for the excited states of the two coinage metals. There is still very little information on excimer formation in mercury or thallium chemistry, where metallophilic bonding to date has played a minor role. By contrast, as mentioned above, the earliest examples of excimer formation by metal complexes were found with platinum(II), where side‐on Pt⋅⋅⋅Pt contacts are formed linking a pair of square‐planar‐coordinated metal atoms thus providing the precursors for excimer formation.

## Conflict of interest

The authors declare no conflict of interest.

## Biographical Information

*Hubert Schmidbaur is Professor Emeritus at the Technical University of Munich (TUM)*, *Germany. After his studies of chemistry at the University of Munich (LMU) he held positions at the universities of Marburg and Würzburg and had numerous appointments abroad. Following work in diverse fields including the chemistry of silicon and phosphorus, π‐complexes of main group elements, the bioinorganic chemistry of magnesium and others, he has dedicated much of his research to the chemistry of gold, for which he received many prestigious awards*.

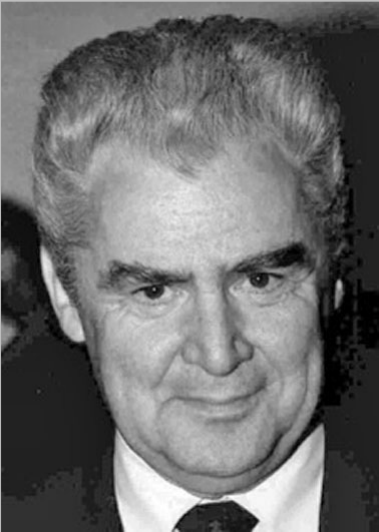

## Biographical Information

*Helgard Raubenheimer is Professor Emeritus at the University of Stellenbosch (SUN), South Africa. He studied chemistry at SUN and later held a chair at the Rand Africaans University in Johannesburg before he returned to SUN as Head of the Department of Chemistry and Polymer Sciences. He spent sabbaticals with E. O. Fischer and H. Schmidbaur at TUM and with D. Seebach at ETH Zürich. His research focused on carbene complexes of the transition metal elements, concentrating in recent years mainly on gold chemistry, which earned him honors in South Africa and abroad*.

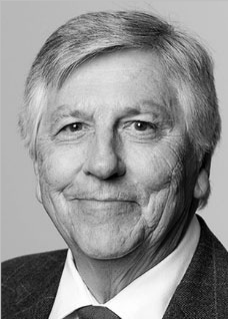

## Supporting information

As a service to our authors and readers, this journal provides supporting information supplied by the authors. Such materials are peer reviewed and may be re‐organized for online delivery, but are not copy‐edited or typeset. Technical support issues arising from supporting information (other than missing files) should be addressed to the authors.

Supplementary
