# Supplementary material for: Excimer and Exciplex Formation in Gold(I) Complexes Preconditioned by Aurophilic Interactions
Source: Angew Chem Int Ed Engl. 2020 Jun 8;59(35):14748–71. doi: 10.1002/anie.201916255 (PMC7496071; doi:10.1002/anie.201916255)
Supplement: Supplementary file 1 — Supplementary [file ANIE-59-14748-s001.pdf]

Angewandte Chemie
Eine Zeitschrift der Gesellschaft Deutscher Chemiker

Supporting Information

Excimer and Exciplex Formation in Gold(I) Complexes Preconditioned by Aurophilic Interactions

Hubert Schmidbaur and Helgard G. Raubenheimer**

anie_201916255_sm_miscellaneous_information.pdf

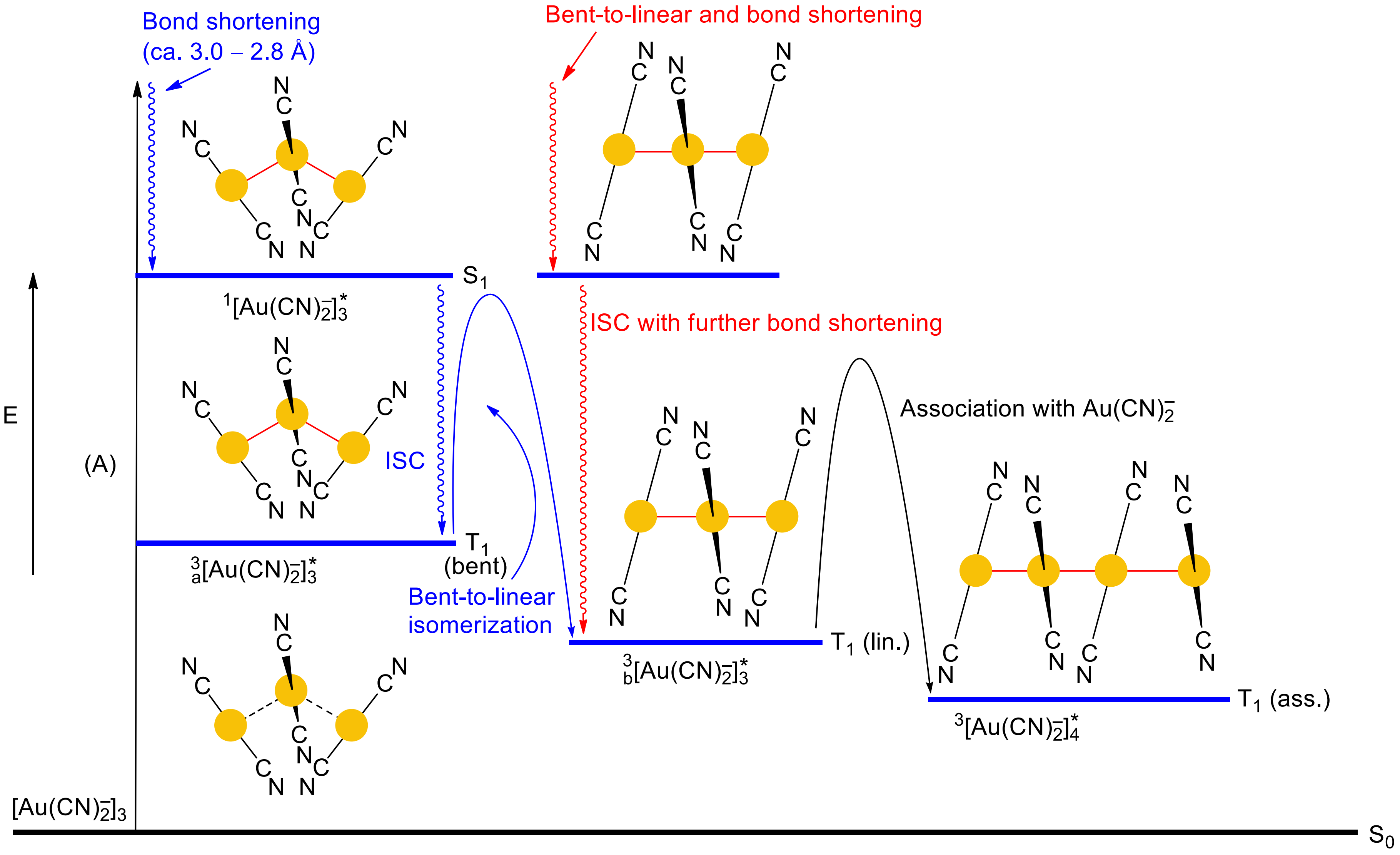

Scheme S1. Structural changes of $[Au(CN)_2^-]_3$ in aqueous solution; blue: [109, 110]; red (where different): [108, 111–113].
